# A genome-wide gain-of-function screen identifies CDKN2C as a HBV host factor

**DOI:** 10.1038/s41467-020-16517-w

**Published:** 2020-06-01

**Authors:** Carla Eller, Laura Heydmann, Che C. Colpitts, Houssein El Saghire, Federica Piccioni, Frank Jühling, Karim Majzoub, Caroline Pons, Charlotte Bach, Julie Lucifora, Joachim Lupberger, Michael Nassal, Glenn S. Cowley, Naoto Fujiwara, Sen-Yung Hsieh, Yujin Hoshida, Emanuele Felli, Patrick Pessaux, Camille Sureau, Catherine Schuster, David E. Root, Eloi R. Verrier, Thomas F. Baumert

**Affiliations:** 1 Université de Strasbourg, Inserm, Institut de Recherche sur les Maladies Virales et Hépatiques UMR_S1110, F-67000, Strasbourg, France; 2 Department of Biomedical and Molecular Sciences, Queen’s University, Kingston, ON Canada; 3 Broad Institute of Massachusetts Institute of Technology and Harvard, Cambridge, MA USA; 4 Inserm, U1052, Cancer Research Center of Lyon (CRCL), Université de Lyon (UCBL1), CNRS UMR_5286, Centre Léon Bérard, Lyon, France; 5 Department of Internal Medicine II/Molecular Biology, University Hospital Freiburg, Freiburg, Germany; 6 Liver Tumor Translational Research Program, Simmons Comprehensive Cancer Center, Division of Digestive and Liver Diseases, Department of Internal Medicine, University of Texas Southwestern Medical Center, Dallas, TX USA; 7 Department of Gastroenterology and Hepatology, Chang Gung Memorial Hospital, Taipei, Taiwan; 8 Institut Hospitalo-Universitaire, Pôle Hépato-digestif, Nouvel Hôpital Civil, 67000 Strasbourg, France; 9 Laboratoire de Virologie Moléculaire, INTS, Paris, France; 10 Institut Universitaire de France (IUF), Paris, France

**Keywords:** Functional genomics, Hepatitis B virus, Viral hepatitis

## Abstract

Chronic HBV infection is a major cause of liver disease and cancer worldwide. Approaches for cure are lacking, and the knowledge of virus-host interactions is still limited. Here, we perform a genome-wide gain-of-function screen using a poorly permissive hepatoma cell line to uncover host factors enhancing HBV infection. Validation studies in primary human hepatocytes identified *CDKN2C* as an important host factor for HBV replication. *CDKN2C* is overexpressed in highly permissive cells and HBV-infected patients. Mechanistic studies show a role for *CDKN2C* in inducing cell cycle G1 arrest through inhibition of CDK4/6 associated with the upregulation of HBV transcription enhancers. A correlation between *CDKN2C* expression and disease progression in HBV-infected patients suggests a role in HBV-induced liver disease. Taken together, we identify a previously undiscovered clinically relevant HBV host factor, allowing the development of improved infectious model systems for drug discovery and the study of the HBV life cycle.

## Abstract (web summary)

Here the authors perform a gain-of-function screen and identify CDKN2C as a host factor for HBV replication, inducing cell cycle arrest and expression of HBV transcription enhancers. CDKN2C expression correlates with disease progression suggesting a potential role in HBV-induced liver disease.

## Introduction

Chronic infection by hepatitis B virus (HBV) is a major health problem and the leading cause of hepatocellular carcinoma (HCC) worldwide^1^. The global HBV burden persists despite the availability of an effective preventative vaccine, and it is estimated that HBV chronically infects 250 million people. While current therapies based on nucleot(s)ide analogs (NUCs) suppress viral replication and reduce progression of liver disease, treatment is lifelong and viral cure is extremely rare^2^. Different curative strategies are urgently needed to address this global medical burden.

HBV is a small enveloped DNA virus in the *Hepadnaviridae* family^3^. The HBV surface antigen (HBsAg) mediates entry of the virus into hepatocytes via primary low-affinity interactions with heparan sulfate proteoglycans^4–6^ and secondary specific binding to the sodium taurocholate cotransporting polypeptide (NTCP)^7,8^, ultimately leading to fusion and release of the viral capsid into the cytoplasm. The capsid delivers the viral genome to the nucleus, where HBV relaxed circular DNA (rcDNA) is converted into episomal covalently closed circular DNA (cccDNA), in a process thought to be mediated by host DNA repair enzymes, such as tyrosyl-DNA-phosphodiesterase 2^9^ and DNA Polymerase kappa^10^. The cccDNA is the reservoir for viral persistence and serves as a template for all viral transcripts. cccDNA levels are not affected by the NUC-based treatments targeting the viral reverse transcriptase, which converts viral pregenomic RNA (pgRNA) into de novo genomic DNA, within newly formed nucleocapsids prior to virion budding^11^.

Currently available drugs for the treatment of chronic HBV infection, such as NUCs, are direct-acting antivirals and allow the suppression of viral replication, but viral cure is rarely achieved. Innovative therapeutic strategies, such as host-targeting agents (HTAs), have emerged as novel candidates for the treatment of viral infections, including hepatotropic viruses^12–15^. However, this strategy requires a comprehensive understanding of virus–host interactions at the molecular level. In the context of HBV infection, the limited access to robust infection models has restrained for a long time the characterization of host factors involved in the viral entry process. The discovery of NTCP as a receptor for HBV has allowed the development of cell culture models suitable for the study of the full life cycle^7,16^. Indeed, exogenous expression of NTCP in human hepatoma cell lines (such as HepG2 and Huh7) confers susceptibility to HBV infection. However, NTCP-overexpressing Huh7 cells remain poorly permissive to HBV infection but support infection by hepatitis D virus (HDV), an HBV-satellite virus carrying HBV envelope proteins^16^. This suggests that after HBV entry, additional key factors are still limiting in these cells. Therefore, we hypothesized that characterization of differences between the two cell lines should allow the identification of previously undiscovered HBV host factors. Discovery of such host factors in human hepatoma cells would open avenues to develop new infection models, such as immunocompetent transgenic animal models that are fully susceptible to HBV. Indeed, a previous study suggests that the limited ability of HBV to replicate in mouse cells is caused by the lack of a host cell-dependency factor^17^. Here we perform a genome-wide gain-of-function screen using a weakly permissive NTCP-overexpressing Huh7-derived cell line termed Huh-106 cells^5^ and a genome-scale lentiviral open reading frame (ORF) library^18^, aiming to uncover HBV-related host-dependency factors. We expect that the identification of these previously undiscovered HBV factors will facilitate the development of improved infectious cell culture systems for the identification of innovative antiviral molecules.

## Results

### A high-throughput screening strategy for HBV host factors

**Fig. 1:**
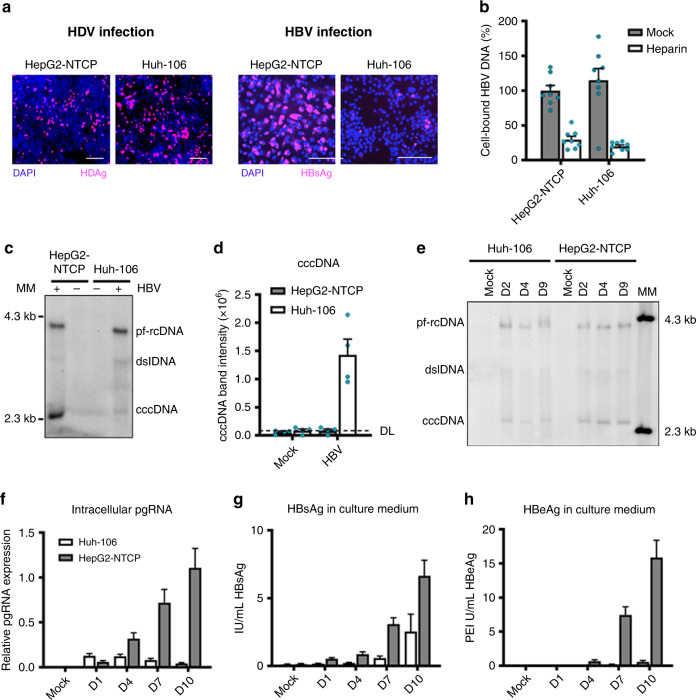
Huh-106 are less permissive to HBV infection than HepG2-NTCP. **a** HBV and HDV infection of HepG2-NTCP and Huh-106 cells and detection of HBsAg and HDAg by IF after 10 dpi. One representative experiment is shown. Scale bars: 100 µm. **b** Binding of HBV particles to HepG2-NTCP and Huh-106 cells. Results are expressed as means +/− SEM bound HBV genome copies (%) from three independent experiments (*n* = 8). **c** Comparison of HBV cccDNA levels in HepG2-NTCP and Huh-106 cells detected by Southern blot. Protein-free relaxed circular DNA (pf-rcDNA), double stranded linear DNA (dsl DNA), and covalently closed circular DNA (cccDNA) are indicated. One representative experiment is shown. **d** Quantification of cccDNA band intensity. Dashed line indicates the detection limit (DL). Results are expressed as means +/− SEM 10^6^ band intensity (arbitrary units) from 4 independent experiments. **e** Time course experiment of HBV infection in Huh-106 and HepG2-NTCP. DNA was extracted from cells 2 (D2), 4 (D4), or 9 (Mock, D9) days post HBV infection and detected by Southern blot. Bands of pf-rcDNA, dsl DNA, and cccDNA were identified using a molecular marker (MM). One experiment is out of three shown. Quantification of cccDNA band intensities in Fig. S5a. **f**–**h** Quantification of intracellular pgRNA by qRT-PCR (**f**) and secreted HBsAg (**g**) and HBeAg (**h**) by CLIA in Huh-106 and HepG2-NTCP cells 1 (D1), 4 (D4), 7 (D7), or 10 (Mock, D10) days post HBV infection. **f** Results are expressed as means +/− SEM relative pgRNA expression from four experiments (*n* = 13). **g** Results are expressed as means +/− SEM IU/mL HBsAg from 4 experiments (*n* = 12). **h** Results are expressed as means +/− SEM PEI U/mL HBeAg from 4 experiments (*n* = 12). MM molecular marker. Source data are provided as a Source Data file.

To characterize HBV infection in different hepatoma cell lines, we compared the susceptibility of two NTCP-overexpressing cell lines (Huh7-derived Huh-106^5^ and HepG2-NTCP) to HBV and HDV infection. Both cell lines were similarly susceptible to HDV infection, suggesting equivalent virus entry in both cell lines (Fig. 1a). However, in contrast to HepG2-NTCP cells, Huh-106 cells appear poorly permissive to HBV infection (Fig. 1a), despite their ability to bind HBV particles (Fig. 1b). Furthermore, Huh-106 cells support the conversion of incoming HBV rcDNA to cccDNA, although to a much lesser extent than HepG2-NTCP cells (Fig. 1c, d). Interestingly, the kinetics of cccDNA formation are similar in both cell lines (Fig. 1e). Moreover, quantification of intracellular pgRNA and secreted antigens (HBsAg and hepatitis B e antigen (HBeAg)) during the course of infection revealed a severe restriction of the HBV life cycle in Huh-106 cells at different steps (Fig. 1f–h). Taken together, these findings suggest that HBV infection is constrained in Huh-106 cells in a step between NTCP-mediated entry and cccDNA-mediated transcription.

**Fig. 2:**
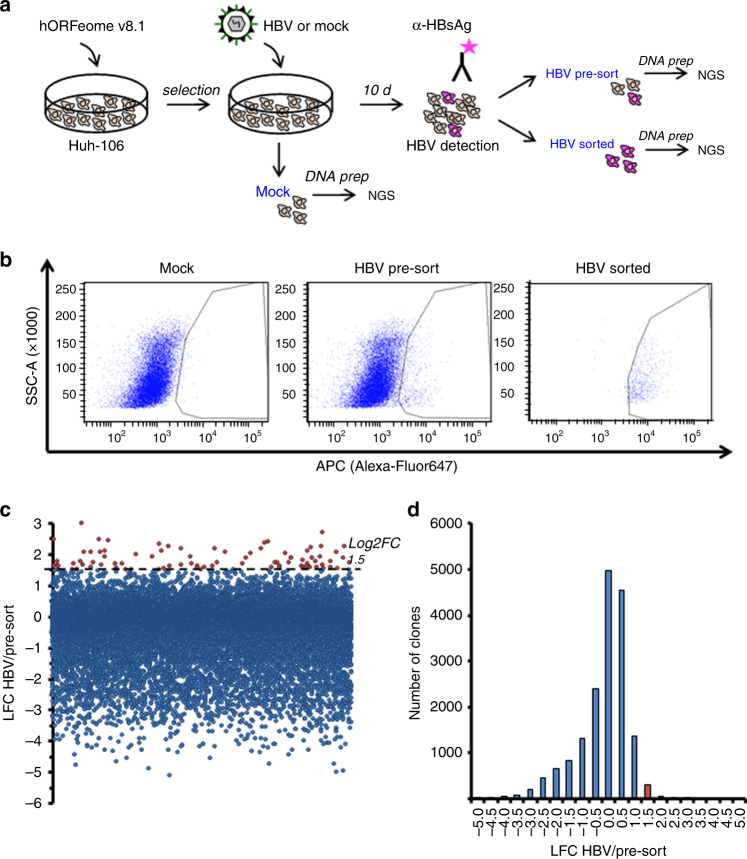
Gain-of-function (GOF) screen in Huh-106 cells for the identification of HBV host factors. **a** Schematic workflow of GOF screen. **b** FACS for HBsAg-positive cells in Huh-106 transduced with an ORF library (hORFeome v8.1) 10 days after HBV infection (HBV pre-sort). Flow cytometric analysis of uninfected cells as gating control (Mock) and of the HBsAg-positive sorted population as sorting control (HBV sorted). **c**, **d** Primary screen candidates. ORFs with Log2FC > 1.5 were selected for validation. Source data are provided as a Source Data file.

Assuming that this restriction is due to the lack of key host factor(s) for HBV infection, we pursued a functional genomics approach to screen for factors that increase the susceptibility of Huh-106 cells to HBV infection. To this end, we performed a gain-of-function screen for HBV infection using Huh-106 cells and a genome-scale lentiviral expression library of >16,000 human ORFs^18^. Huh-106 cells were first transduced with the lentiviral hORFeome V8.1^18^ and then inoculated with HBV (Fig. 2a). Sorting for HBsAg-positive cells by fluorescence-activated cell sorting (FACS) 10 days postinfection (dpi) allowed the collection of HBV-infected cells only (HBV sorted) for subsequent analysis to identify factors conferring susceptibility to HBV infection. Using Illumina next-generation sequencing and deconvolution using PoolQ, we compared the infected pool of cells (HBV sorted, Fig. 2a, b) to the control population (HBV pre-sort, Fig. 2a, b) to determine which ORFs were enriched in HBsAg-positive cells. Candidate HBV host factors were identified based on an enrichment threshold of log2 fold change (Log2FC) >1.5 (Fig. 2c, d). ﻿Following an algorithm based on liver expression and the number of sequences per candidate to further filter the list (see “Methods”), 47 candidate genes were selected for validation (Supplementary Table 1). Among them was *HNF4A*, a gene encoding a transcription factor previously known to enhance HBV replication^19^, supporting the ability of our screen to identify HBV host factors. Interestingly, another transcription factor stimulating HBV replication, *HLF*^20^, scored a Log2FC = 1.49 just below the selection threshold. The remaining candidates therefore represent a list of putative new factors for HBV infection for further validation and study.

### Cyclin-dependent kinase inhibitor 2C (CDKN2C) is a HBV host factor highly expressed in HepG2 cells

**Fig. 3:**
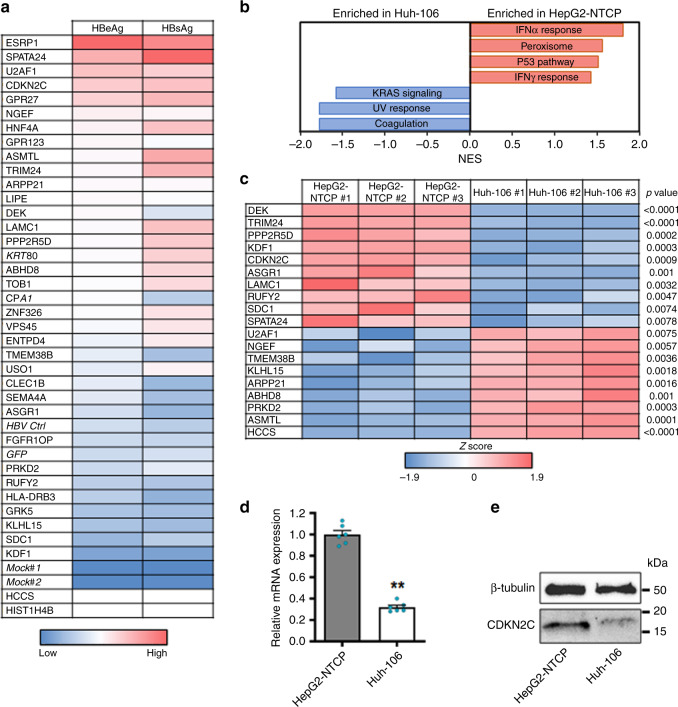
CDKN2C is differentially expressed in HepG2-NTCP and Huh-106 cells. **a** Heatmap of candidate validation. Huh-106 cells were transduced with the indicated ORF and infected with HBV. HBV infection was assessed at 10 dpi by CLIA quantification of secreted HBeAg and HBsAg. Results are expressed as means concentration of secreted HBeAg or HBsAg from 1 experiment (*n* = 2). Genes in italic (*KRT80* and *CPA1*) correspond to negative controls, which were not identified as candidates from the primary screen. Mock#1 and Mock#2: uninfected HepG2-NTCP cells. HBV ctrl: non-transduced HBV-infected HepG2-cells. GFP: GFP-transduced HBV-infected HepG2-NTCP cells. **b**, **c** Microarray for comparison of gene expression in HepG2-NTCP and Huh-106 cells. Analysis of differentially expressed pathways (**b**) and candidate host factors from the primary screen through *Z* score transformation (**c**) are presented. **d**, **e** CDKN2C is upregulated in HepG2-NTCP compared to Huh-106 cells. **d** *CDKN2C* mRNA expression in HepG2-NTCP and Huh-106 cells quantified by qRT-PCR. Results are expressed as means +/− SEM *CDKN2C* relative expression compared to HepG2-NTCP (set to 1) from 3 independent experiments (*n* = 6). **e** Endogenous CDKN2C protein expression in HepG2-NTCP and Huh-106 cells detected by western blot. One representative experiment is shown. **p < 0.01 (two-tailed Mann–Whitney *U* test). Source data are provided as a Source Data file.

To validate the candidate host factors identified above, we individually overexpressed the candidate ORFs in Huh-106 cells before infection with HBV for 10 days. Of the 47 identified ORFs, 35 were evaluated (see “Methods”), along with lentiviruses encoding *GFP*, *KRT80*, and *CPA1* as negative controls (Supplementary Table 1). HBV infection was assessed by quantification of secreted HBV antigens in the cell culture supernatant of infected cells, indicating increased HBV infection versus controls for a majority of the candidates. Several had large effects on both secreted HBeAg and HBsAg, including the top scorers *ESRP1*, *SPATA24*, *U2AF1*, *CDKN2C*, and *GPR27* (Fig. 3a, Supplementary Fig. 1). Importantly, the top candidate ESRP1 was not detected at the protein level in our systems (data not shown), suggesting a non-physiological effect on HBV infection. However, this construct was used as a technical positive control in further experiments. To systematically identify genes that are differentially expressed in the studied cell lines, we performed transcriptomic analyses using microarrays for gene expression profiling in HepG2-NTCP and Huh-106 cells. Pathway enrichment analysis identified a small number of signaling pathways that exhibited significantly different expression patterns between the two cell lines, although the vast majority of pathways were similarly expressed (Fig. 3b). Notably, interferon-α response gene expression was higher in HepG2 cells, consistent with previous observations that HepG2 cells are more competent for mounting an efficient innate immune response following viral infection compared to Huh7-derived cells^21,22^.

**Fig. 4:**
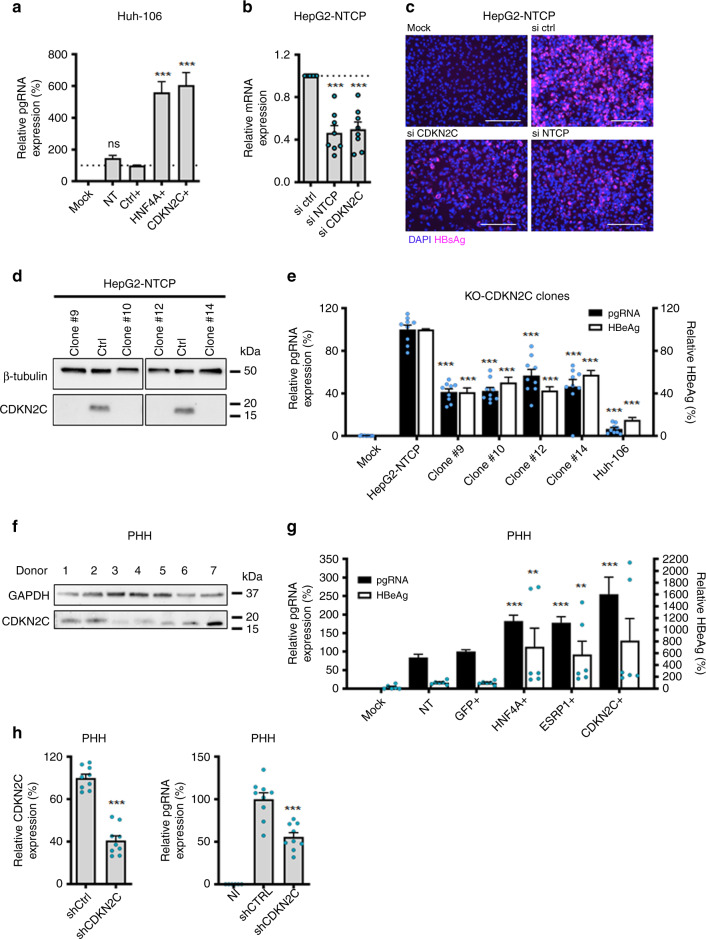
CDKN2C is a HBV host factor. **a** Individual ORF overexpression in Huh-106 and HBV infection 3 days after transduction. Detection of HBV pgRNA by qRT-PCR 10 dpi. Results are expressed as means +/− SEM relative pgRNA expression (%) compared to ctrl (set as 100%) from 8 independent experiments (*n* = 21). **b**, **c** siRNA transfection of HepG2-NTCP cells. **b** mRNA. Results are expressed as means +/− SEM relative expression compared to si ctrl (set to 1) from 4 independent experiments (*n* = 8). **c** HBV infection after silencing was detected by IF 10 dpi. Scale bars: 100 µm. **d** Production of *CDKN2C* knockout cell lines. CDKN2C expression was controlled by western blot for in HepG2-NTCP (ctrl) and KO-CDKN2C clones. **e** HBV infection of HepG2-NTCP, KO-CDKN2C clones, and Huh-106. HBV infection was assessed at 10 dpi by pgRNA qRT-PCR (black) and quantification of secreted HBeAg (white). Results are expressed as means +/− SEM % HBV infection compared to HepG2-NTCP (set as 100%) from 3 independent experiments (*n* = 9 for pgRNA and *n* = 12 for HBe CLIA). **f** Detection of endogenous CDKN2C expression in PHH from 7 donors. One experiment is shown. **g** Validation studies in PHH from 3 different donors transduced with ORF lentivirus for 3 days and infected with HBV. HBV markers (pgRNA, black; HBeAg, white) were detected 10 dpi. Results are expressed as means +/− SEM % HBV infection compared to ctrl (GFP) (set to 100%) from 3 independent experiments (*n* = 12 for pgRNA; *n* = 6 for HBeAg). **h** PHH from 3 donors were transduced with lentiviruses containing CDKN2C-targeting shRNA or non-targeting shRNA control (sh ctrl). Silencing efficacy was assessed by qRT-PCR. Results are expressed as means +/− SEM % gene expression compared to sh ctrl (set to 100%) from 3 independent experiments (*n* = 9). PHH were then infected with HBV and HBV infection was assessed by pgRNA qRT-PCR 8 dpi. Results are expressed as means +/− SEM relative pgRNA expression compared to sh ctrl (set to 100%) from 3 independent experiments (*n* = 9). **p < 0.01; ***p < 0.001 (two-tailed Mann–Whitney *U* test). Source data are provided as a Source Data file.

Comparing the expression of primary screen candidate genes from the microarray data, we identified *CDKN2C* and *SPATA24* as highly expressed genes in HepG2-NTCP versus Huh-106 cells (Fig. 3c). Given the specific previously annotated function of SPATA24/T6441 in spermiogenesis^23^, we focused instead on *CDKN2C* for further characterization. The higher expression of *CDKN2C* in HepG2-NTCP versus Huh-106 cells was confirmed by quantitative PCR (qPCR) and western blot (Fig. 3d, e). The involvement of *CDKN2C* in HBV infection in Huh-106 cells was confirmed by a sixfold increase in viral pgRNA levels following overexpression of *CDKN2C* when compared to the empty control vector (Fig. 4a).

Taking advantage of high infection levels in HepG2-NTCP cells, we aimed to confirm the phenotypic effect of *CDKN2C* on HBV infection by a loss-of-function approach, using small interfering RNA (siRNA) specifically targeting *CDKN2C* or *SLC10A1* (the gene encoding the HBV receptor NTCP) in susceptible HepG2-NTCP cells, as shown in Fig. 4b, c. We observed a marked decrease in HBV infection in cells with silenced *CDKN2C* or *SLC10A1* expression. To rule out off-target effects, we used CRISPR-Cas9 to generate and clonally select four independent HepG2-NTCP CDKN2C knockout (KO) cell lines (Fig. 4d). Functional analysis confirmed a marked decrease in both HBV pgRNA and secreted HBe antigen levels in HepG2-NTCP KO-CDKN2C cells compared to naive HepG2-NTCP cells (Fig. 4e). Finally, to validate the relevance of *CDKN2C* in a physiological model, we investigated CDKN2C–HBV interactions in primary human hepatocytes (PHHs), the natural target cells for HBV infection, which express the protein at varying levels comparable to HepG2-NTCP cells (Fig. 4f). Consistent with our previous observations, the overexpression of *HNF4A* and *CDKN2C* in PHHs resulted in a significant and marked increase in HBV infection (Fig. 4g). Moreover, the silencing of *CDKN2C* expression using target-specific short hairpin RNA (shRNA) induced a significant and robust decrease in HBV infection (Fig. 4h). Taken together, our data support a role for *CDKN2C* in HBV infection. Therefore, the differential expression of this gene between the two cell lines suggests that a lack of *CDKN2C* expression may contribute to the limited susceptibility of Huh-106 cells to HBV infection.

### CDKN2C stimulates HBV cccDNA-mediated transcription

**Fig. 5:**
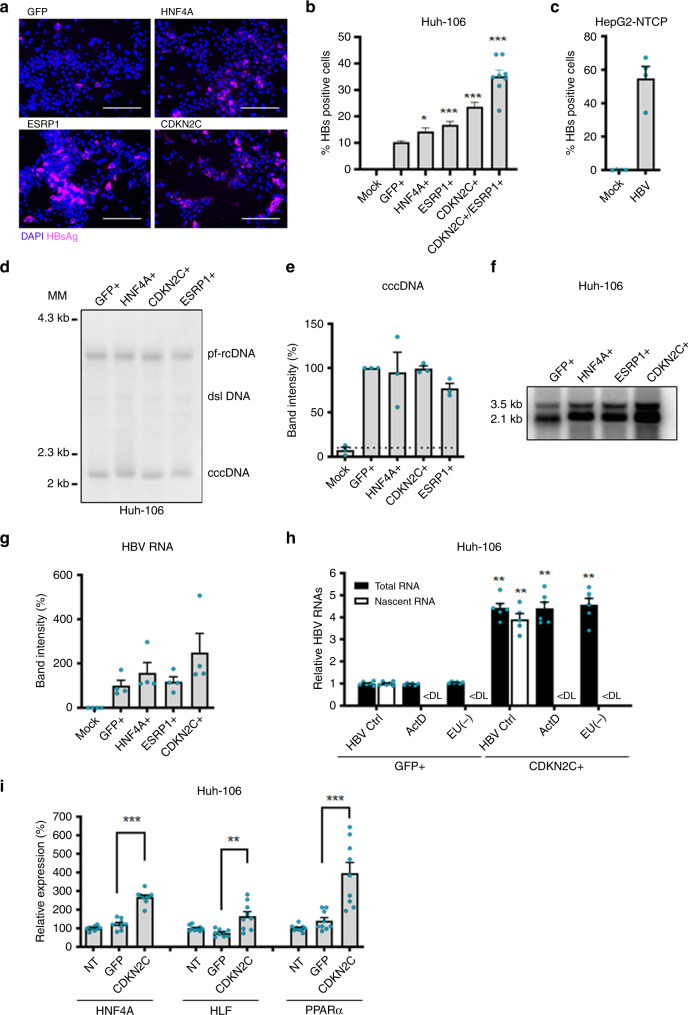
CDKN2C stimulates HBV cccDNA-mediated transcription. **a**, **b**, **d**–**g** Validation studies in Huh-106 overexpressing individual ORFs and infected with HBV for 10 days. **a** Detection of HBsAg by IF. Scale bars: 100 µm. **b** Flow cytometric analysis for quantification of HBsAg-positive cells. Results are expressed as means +/− SEM % HBsAg-positive cells compared to GFP from 5 independent experiments (*n* = 13, *n* = 11 for HNF4A) and 3 independent experiments (*n* = 8) for CDKN2C + ESRP1 **c** Flow cytometric analysis for quantification of HBsAg-positive cells in HBV-infected HepG2-NTCP cells. Results are expressed as means +/− SEM % HBsAg-positive cells from 4 independent experiments (*n* = 4). **d**, **e** Detection of HBV DNAs by Southern blot in transduced and HBV-infected Huh-106 4 dpi. **d** Southern blot with the indicated bands of HBV pf-rcDNA, dsl HBV DNA, and HBV cccDNA. One representative experiment is shown. **e** Quantification of cccDNA. Results are expressed as means +/− SEM % band intensity compared to GFP (set to 100%) from 3 independent experiments (*n* = 2). **f** Detection of HBV RNAs by northern blot. The pgRNA (3.5 kb) and surface mRNAs of 2.1 to 2.4 kb (2.1 kb) are detected. One representative experiment is shown. **g** Quantification of HBV RNA band intensity. Results are expressed as means +/− SEM % band intensity compared to GFP (set to 100%) from 4 independent experiments. **h** Analysis of nascent HBV RNA synthesis. Quantification of total HBV RNAs (4 dpi) and nascent HBV RNAs (d4pi, 120 min) in Huh-106 cells overexpressing CDKN2C using labeled uridine (EU). Actinomycin D (ActD) was used as negative control. Results are expressed as means +/− SEM % relative HBV RNAs compared to HBV Ctrl (Huh-106 GFP+ set to 1) from 2 independent experiments (*n* = 6). **i** *HNF4a*, *HLF* and *PPARα* mRNA expression in CDKN2C-overexpressing Huh-106 quantified by qRT-PCR. Results are expressed as means +/− SEM % relative HNF4a or HLF or PPARα expression compared to Mock (set to 100%) from 3 independent experiments (*n* = 9) *p < 0.05; **p < 0.01; ***p < 0.001 (two-tailed Mann–Whitney *U* test). MM molecular marker. Source data are provided as a Source Data file.

To address the mechanism by which *CDKN2C* contributes to HBV infection, we performed additional experiments using alternative read-outs to identify the steps of the viral life cycle that may be affected by *CDKN2C* expression. Transduction efficacy was assessed by quantification of green fluorescent protein (GFP) expression in HBV-infected *GFP*-transduced cells after 10 days (Supplementary Fig. 2). Detection of intracellular HBsAg by immunofluorescence (IF; Fig. 5a) and its quantification by flow cytometric analysis (Fig. 5b) revealed a significant increase in HBV infection levels in Huh-106 cells overexpressing *HNF4A*, *ESRP1*, and *CDKN2C*. Notably, co-overexpression of *CDKN2C* and *ESRP1* leads to an even higher percentage of HBsAg-positive cells (Fig. 5b), suggesting that the two factors affect HBV infection through independent pathways. Interestingly, overexpression of both factors in Huh-106 cells markedly increased HBV infection but failed to reach levels observed in HepG2-NTCP cells (Fig. 5b, c), suggesting the existence of additional differentially expressed factors in the two cell lines. To determine the step of the HBV life cycle affected by *CDKN2C* expression, we detected HBV DNA genome intermediates by Southern blot and HBV RNA levels by northern blot. As shown in Fig. 5d, e, no marked change in HBV cccDNA levels was observed when *CDKN2C* was overexpressed, suggesting no effect on HBV replication before cccDNA formation. Detection of viral RNAs by Northern blot revealed increased HBV RNA levels in cells overexpressing *HNF4A* and *CDKN2C* compared to *GFP*-overexpressing cells (Fig. 5f, g). To determine whether CDKN2C has a direct effect on HBV RNA formation, we quantified nascent HBV RNAs using labeled uridine. Huh-106 cells overexpressing *CDKN2C* displayed a threefold increased level of newly synthesized HBV RNA (Fig. 5h). This suggests a role for CDKN2C in cccDNA-mediated transcription of HBV RNAs. To investigate whether the role of CDKN2C in transcription of HBV RNAs is linked to previously described HBV host factors, we quantified the expression of *HNF4A*, *HLF*, and *PPARA*, known to enhance HBV transcription^19,20^. Interestingly, *CDKN2C* overexpression in Huh-106 resulted in upregulation of the expression of three HBV transcription factors (Fig. 5i). Taken together, our results suggest that *CDKN2C* expression enhances transcription of HBV RNAs through the upregulation of HBV-related transcription factors.

### Enhanced supernatant infectivity of transduced HepAD38 cells

**Fig. 6:**
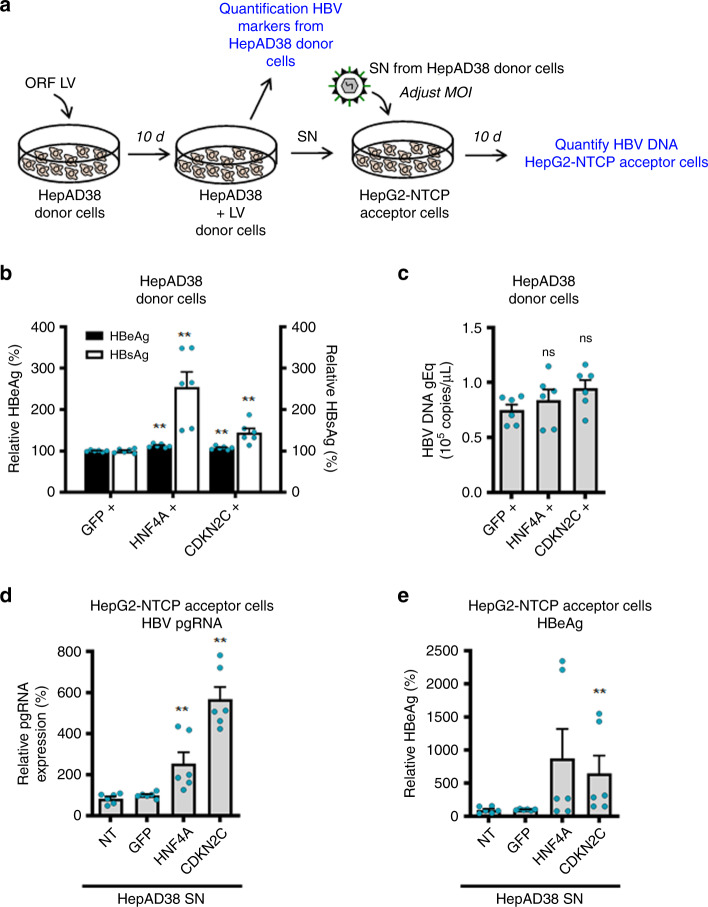
*CDKN2C* overexpression results in enhanced infectivity of supernatants of transduced HepAD38 cells. **a** Schematic workflow of experiments. HepAD38 cells in production medium (Donor cells) were non-transduced (NT) or transduced with ORF lentivirus for 10 days. **b**, **c** Supernatant (SN) from HepAD38 donor cells was harvested and HBV markers were quantified from SN. **b** HBeAg and HBsAg secretion was quantified by CLIA. Results are expressed as means +/− SEM % secreted HBeAg or % secreted HBsAg compared to NT (set to 100%) from 3 independent experiments (*n* = 6). **c** HBV DNA level in the supernatant was determined by qPCR. Results are expressed as means +/− SEM HBV DNA genome equivalents from 3 independent experiments (*n* = 6). **d**, **e** HepG2-NTCP (Acceptor cells) were infected with adjusted MOI from supernatant from HepAD38 donor cells. **d** HBV pgRNA expression was quantified by qRT-PCR 10 dpi. Results are expressed as means +/− SEM % relative pgRNA expression compared to NT (set at 100%) from 3 independent experiments (*n* = 6). **e** HBeAg secretion was quantified by CLIA 10 dpi. Results are expressed as means +/− SEM % relative secreted HBeAg compared to NT (set at 100%) from 3 independent experiments (*n* = 6). **p < 0.01 (two-tailed Mann–Whitney *U* test). Source data are provided as a Source Data file.

Since a recent study suggested that HBV virion production was more efficient in quiescent cells^24^, we then investigated whether modification of *CDKN2C* expression modulates the production of virus particles in HBV-expressing cells, and we overexpressed *CDKN2C* and *HNF4A* in HepAD38 donor cells. Ten days after ORF lentivirus transduction, we harvested supernatants and infected HepG2-NTCP acceptor cells with an adjusted multiplicity of infection (MOI) from supernatant from HepAD38 donor cells containing HBV particles (Fig. 6a). While we observed a modest increase in the secretion of HBsAg and HBeAg, *CDKN2C* overexpression in HepAD38 donor cells did not affect the levels of HBV DNA in the cell culture supernatant (Fig. 6b, c). Interestingly, overexpression of *CDKN2C* in HepAD38 increased infection of HepG2-NTCP acceptor cells by about threefold suggesting that the supernatant of *CDKN2C*-transduced HepAD38 cells has a higher infectivity (Fig. 6d, e).

### CDK4/6 inhibitors enhance HBV infection

**Fig. 7:**
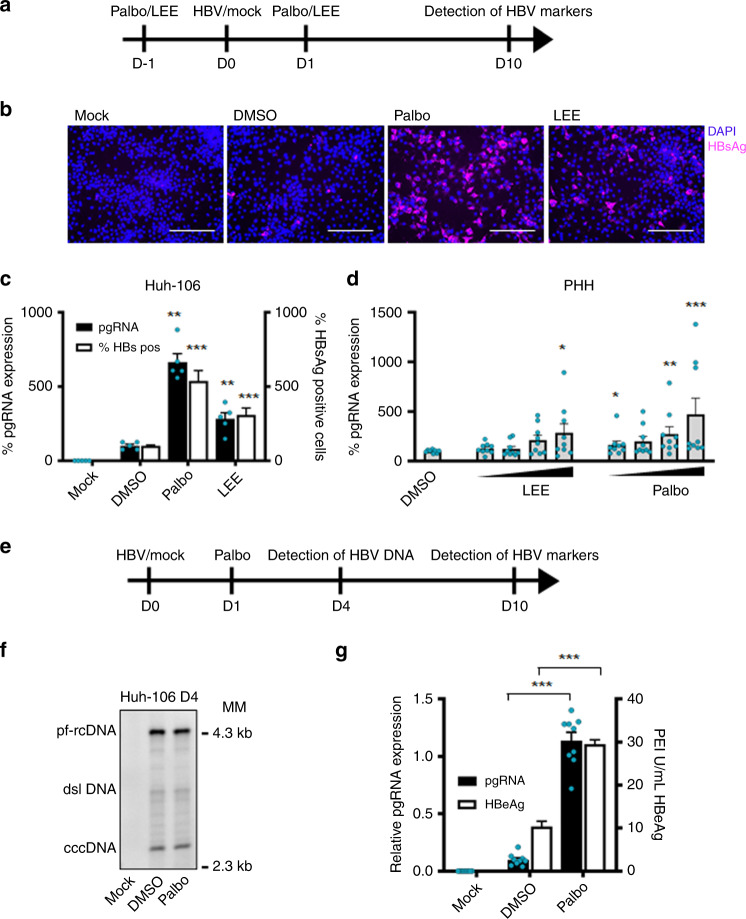
CDKN2C-mediated stimulation of HBV transcription is correlated with a cell cycle arrest. Effect of treatment with CDK4/6 inhibitors Palbociclib (Palbo) and LEE011 (LEE) on HBV infection. **a** Schematic workflow of experiments. **b**–**d** Detection of HBV markers in mock/HBV infected Huh-106 cells or PHHs treated with DMSO or Palbo/LEE before (D−1 to D0) and after (D1 to D10) HBV infection 10 dpi. **b**, **c** Detection of HBV markers 10 dpi in mock-treated of HBV-infected Huh-106 cells treated with DMSO or 100 nM Palbo/LEE. **b** Detection of HBsAg by IF 10 dpi. Scale bars: 100 µm. **c** Quantification of HBV pgRNA by qRT-PCR (black). Quantification of HBsAg-positive cells by flow cytometric analysis (white). Results are expressed as means +/− SEM % HBV infection compared to DMSO (set to 100%) from 3 independent experiments (*n* = 5) for pgRNA and from 4 independent experiments (*n* = 12) for % HBsAg positive. **d** Quantification of HBV pgRNA10 dpi in mock-treated of HBV-infected PHHs treated with DMSO or 1–1000 nM Palbo/LEE. Results are expressed as means +/− SEM % relative pgRNA expression compared to DMSO (set to 100%) from 3 independent donors (*n* = 9). **e** Schematic workflow of experiments. **f**–**h** Treatment of mock/HBV-infected Huh-106 or HepG2-NTCP cells with 0 nM (DMSO) or 100 nM Palbociclib (Palbo) after HBV infection. **f** Detection of HBV DNA by Southern blot in Huh-106 cells 4 dpi. HBV pf-rcDNA and dsl DNA cccDNA bands are indicated. One representative experiment is shown. Quantification of cccDNA bands in Fig. S5b. **g** Detection of HBV markers in Huh-106 10 dpi. Quantification of HBV pgRNA by qRT-PCR (black) and of secreted HBeAg by CLIA (white). Results are expressed as means +/− SEM relative pgRNA expression (pgRNA) or as means +/− SEM PEI U/mL HBeAg from 3 independent experiments (*n* = 9) for pgRNA and from 3 independent experiments (*n* = 12) for HBeAg. *p < 0.05; **p < 0.01; ***p < 0.001 (two-tailed Mann–Whitney *U* test). MM molecular marker. Source data are provided as a Source Data file.

**Fig. 8:**
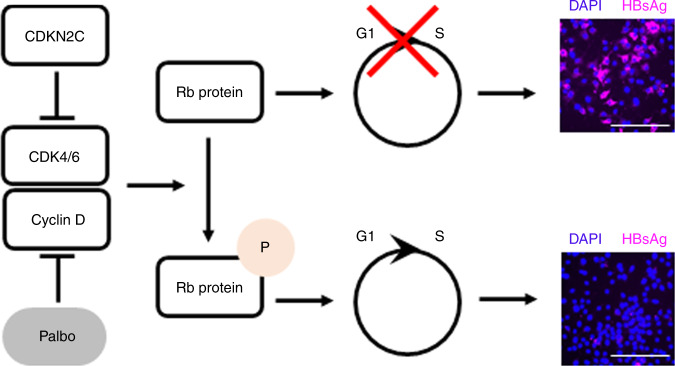
Schematic model of the effect of CDKN2C expression and Palbociclib (Palbo) treatment on HBV infection. CDKN2C and Palbociclib inhibit the CDK4/6 and Cyclin D-mediated phosphorylation of Rb protein, leading to an accumulation of Rb protein in its unphosphorylated state. Unphosphorylated Rb protein induces a cell cycle G1 arrest resulting in increased HBV infection rates. Illustrative HBV infection pictures come from Fig. 6. Scale bars: 100 µm.

*CDKN2C* encodes the CDKN2C, a regulator of G1 cell cycle progression through interaction with cyclin-dependent kinases 4 and 6 (CDK4/6). In fact, overexpression of *CDKN2C* induces G1 cell cycle arrest in Huh-106 cells (Supplementary Fig. 3). To determine whether this known function of CDKN2C is responsible for enhancing HBV infection, we performed functional studies using two clinically studied CDK4/6-specific small molecule inhibitors, Palbociclib^25^ and LEE011^26^. Drug treatment of Huh-7 and Huh-106 cells induced a dose-dependent G1 cell cycle arrest associated with a decrease in cell proliferation (Supplementary Fig. 4a–d), most likely due to drug-induced cytostatic effect associated with the accumulation of cells in G1 phase. At the reference concentration of 100 nM, Palbociclib and LEE011 did not induce major cytotoxic effects as shown by the LDH-Glo cytotoxicity assay (Supplementary Fig. 4b). We then determined HBV infection levels in Huh-106 cells treated with either of the inhibitors before and after HBV infection (Fig. 7a, e). Visualization of intracellular HBsAg revealed a marked increase in HBV infection levels after treatment with Palbociclib or LEE011 (Fig. 7b). Furthermore, quantification of HBV pgRNA and HBsAg-positive cells revealed a significant increase in HBV infection upon both Palbociclib and LEE011 treatment (Fig. 7c). Similar results were obtained in PHHs treated with CDK4/6 inhibitors at different concentrations (1, 10, 100, and 1000 nM) confirming the proviral effect of Palbociclib and LEE011 (Fig. 7d). To investigate whether Palbociclib-mediated enhancement of infection is dependent on HBV entry, we treated HBV infected Huh-106 cells with 100 nM Palbociclib following removal of the HBV inoculum (Fig. 7e). As shown in Fig. 7f and Supplementary Fig. 5, Palbociclib treatment did not affect HBV cccDNA levels, suggesting no effect on the viral entry steps including cccDNA formation. However, pgRNA and secreted HBeAg levels were significantly increased in Palbociclib-treated cells, indicating that CDKs are important for post-entry steps of the viral life cycle (Fig. 7g). Collectively, our data identify *CDKN2C* as a previously undiscovered HBV host factor, most likely acting through inhibition of CDK4/6, triggering a cell cycle G1 arrest and enhancing HBV transcription (Fig. 8).

### *CDKN2C* expression is associated with chronic liver disease

**Fig. 9:**
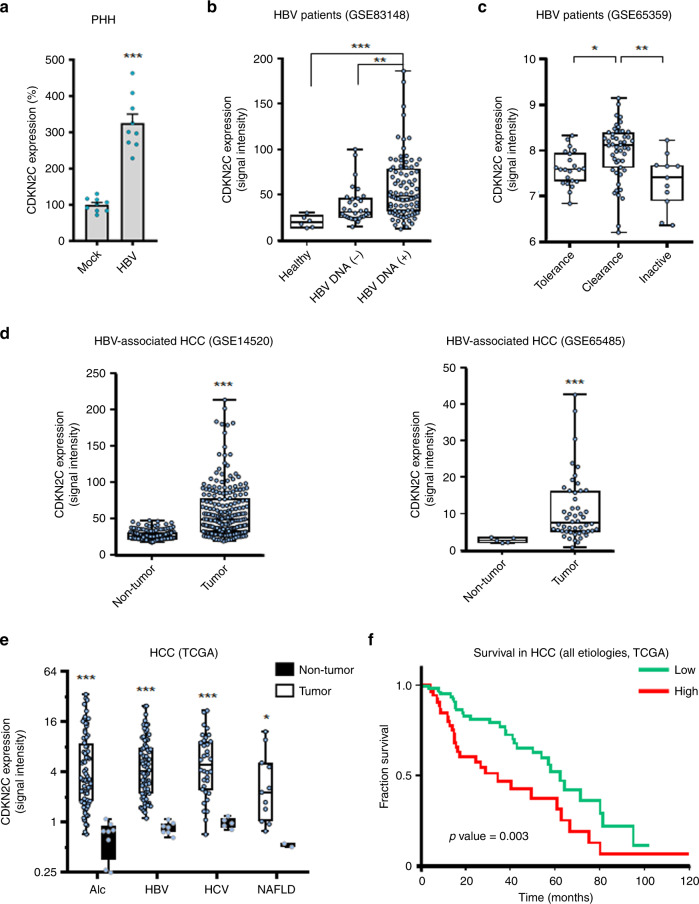
CDKN2C expression is associated with HBV infection, liver disease, and survival in patients. **a** *CDKN2C* mRNA expression in HBV-infected PHH from 3 different donors quantified by qRT-PCR. Results are expressed as means +/− SEM % relative CDKN2C expression compared to Mock (set to 100%) from 3 independent experiments (*n* = 9). **b** *CDKN2C* expression in HBV-infected patients with undetectable (HBV DNA(−), *n* = 32) or detectable (HBV DNA(+), *n* = 90) HBV DNA compared to healthy patients (*n* = 6) (cohorts described in “Methods”). **c** *CDKN2C* expression in HBV-infected patients depending on the stage of virus infection (cohorts described in “Methods”). Tolerance: *n* = 22; Clearance: *n* = 50; Inactive: *n* = 11. **d** *CDKN2C* expression in tumor and adjacent tissues in HCC patients from two independent cohorts (see “Methods”). Non-tumor: *n* = 198; Tumor: *n* = 98 (left panel). Non-tumor: *n* = 5; Tumor: *n* = 50 (right panel). **e** *CDKN2C* expression in tumor and non-tumor (normal) liver tissue from patients with alcoholic liver disease (Alc, Tumor: *n* = 70; Non-tumor: *n* = 8), HBV-infected patients (Tumor: *n* = 76; Non-tumor: *n* = 7), HCV-infected patients (Tumor: *n* = 34; Non-tumor: *n* = 5), and patients with non-alcoholic fatty liver disease (NAFLD, Tumor: *n* = 11; Non-tumor: *n* = 2) extracted from TCGA database as described in “Methods.” **f** Survival analysis for HCC patients with low or high CDKN2C expression (for cohort, see “Methods”). *p < 0.05; **p < 0.01; ***p < 0.001 (**b**, **c**: Kruskal–Wallis *H* test adjusted for multiple comparisons; **d**, **e**: two-tailed Mann–Whitney *U* test). The details of the plots are presented in Supplementary Tables 2 and 3. Source data are provided as a Source Data file.

To assess whether HBV infection directly affects *CDKN2C* expression, we infected PHHs with HBV and evaluated *CDKN2C* gene expression. Interestingly, *CDKN2C* expression was upregulated upon HBV infection (Fig. 9a). In line with this observation, the analysis of *CDKN2C* expression from patient liver tissues retrieved from the Gene Expression Omnibus database revealed an upregulation of *CDKN2C* in patients with active replication compared to patients with undetectable viral load and healthy patients (Fig. 9b). Moreover, a correlation was observed between HBV viral load and *CDKN2C* expression in liver tissues from nine HBV-infected patients (Supplementary Fig. 6a). Finally, *CDKN2C* expression appeared to be modulated in different stages of HBV infection (Fig. 9c). Taken together, these data suggest that HBV infection modulates *CDKN2C* expression in chronically infected patients. To evaluate whether *CDKN2C* expression is associated with the development of virus-induced liver disease, we analyzed *CDKN2C* expression in HBV patients with advanced liver disease and HCC. We first observed that patients with advanced fibrosis (F3) exhibit higher *CDKN2C* mRNA levels compared to patients with F1 or F2 fibrosis *CDKNC2* expression (Supplementary Fig. 6b). Moreover, *CDKN2C* expression was significantly higher in tumor tissues from HBV-derived HCC compared to adjacent tissue (Fig. 9d). To assess the specificity of this correlation, we analyzed *CDKN2C* expression in HCC patients regardless the etiology. *CDKN2C* levels were markedly elevated in the tumor liver tissue of patients chronically infected with hepatitis C virus (HCV) or HBV and patients with alcoholic liver disease (ALD) or non-alcoholic fatty liver disease (NAFLD) as compared with non-tumor tissue (Fig. 9e), suggesting that *CDKN2C* expression is upregulated in HCC in an etiology-independent manner. Finally, higher expression of *CDKN2C* in HCC patients was associated with significantly lower long-term overall survival (Fig. 9f). Taken together, our data suggest that HBV infection modulates *CDKN2C* expression and that *CDKN2C* expression is associated with liver disease progression and poor survival.

## Discussion

Chronic hepatitis B is the most common form of severe viral hepatitis worldwide and a leading cause of HCC. To date, molecular details of HBV–host interactions are not fully understood. Using a functional genomics approach, we identified *CDKN2C* as a previously undiscovered host factor for HBV infection. The functional impact of this finding is confirmed by: (1) a marked increase or decrease in HBV infection after *CDKN2C* overexpression or KO, respectively; (2) an increase in HBV markers following *CDKN2C* overexpression; and (3) a significant proviral effect of CDK4/6 inhibitors correlated with cell cycle G1 arrest. The role of *CDKN2C* as an HBV host factor was identified in a gain-of-function approach combining a cell-based model system^5^ with a genome-scale ORF library^18^. The ability of our screen to discover HBV host factors promoting different steps of the HBV life cycle is supported by the identification of *HNF4A* in the primary screen. *HNF4A* encodes a liver-specific transcription factor, hepatocyte nuclear factor 4 (HNF4), that has been shown to be important for HBV replication by enhancing transcription from the promoters of HBV core^27^, major surface antigen and large surface antigen^19^. Hence, HNF4A is likely to be a key transcription factor that regulates the HBV replication cycle and contributes to hepatotropism^28,29^. Notably, the hepatic leukemia factor (HLF), another transcription factor playing a role in the regulation of the HBV core promoter via interaction with sites other than HNF4^20^, scored with a Log2FC value of 1.49 just below our threshold for selection of candidate host factors. This supports the ability of our screening strategy to detect HBV host factors. Notably, the screen and validation experiments identified *ESRP1* as the top candidate HBV host factor. *ESRP1* encodes a splicing regulator especially involved in a large splicing program critical for the development in mammals^30^. Importantly, ESRP1 protein expression was not detected in our systems, suggesting no or weak expression in hepatocytes. It is, however, likely that the splicing regulation of hepatocyte factors or the virus transcripts themselves (as it has been described, see ref. ^31^) explain the observed effect, even if not physiologically relevant.

While some proviral and antiviral host factors have been described, many aspects of virus–host interactions remain poorly understood. Importantly, the correlation between HBV replication and cell cycle progression has long been a topic of investigation. For instance, in 1979, Aden et al.^32^ demonstrated increased HBV antigen production in non-dividing tumor-derived cells with integrated HBV DNA sequences. Similar observations were made in an HBV-transfected hepatoma-derived cell line^33^. Later, HBV replication was found to be inversely correlated to cellular DNA synthesis and to be enhanced in quiescent hepatocytes^34^. In fact, effective in vitro infection with HBV requires the presence of dimethyl sulfoxide (DMSO), known to enhance and prolong HBV infection by several mechanisms^16,35^ and to decrease cell proliferation^36^. It has been previously described that HBV preferentially infects resting cells and that the virus is able to deregulate the infected cell cycle to favor its replication^37,38^. However, it remains unclear which host factors are involved in that process and whether cells arrested in G0/G1 or G2/M phase are more prone to HBV infection. Our data support the hypothesis that G1 cell cycle arrest is favorable for HBV replication and that CDKN2C is a key host factor mediating this virus–host interaction. A comparison of the proliferative ability of HepG2 cells with that of HepG2.2.15 (constitutively expressing HBV from integrated viral DNA) indicated that HBV induces a G1 phase arrest^37^. It has also been shown in PHH that HBV arrests infected cells in the G2/M phase and replicates more favorably during this cell cycle phase^38^. In eukaryotic cells, CDKs are key components of cell cycle regulation machinery. They form complexes with cyclins to control the transition through cell cycle phases and therefore allow cell division of healthy cells^39^. ﻿Interactions of HBV with certain CDKs have been shown. For example, CDK2 is involved in the phosphorylation of HBcAg and might be incorporated into viral capsids^40^. Moreover, inhibitors of CDKs have been shown to modulate HBV infection with different outcomes. On the one hand, KO or inhibition of CDK2 enhances HBV replication by phosphorylation and deactivation of the host restriction factor SAMHD1^41^. On the other hand, the CDK9 inhibitor FIT039 prevents replication of HBV and other DNA viruses and is under consideration as an antiviral candidate against HBV^42,43^. These data suggest a link between the CDK–cyclin pathway and the HBV life cycle. However, the key components and mechanisms remain unclear.

Here we identify CDK4/6 as additional players in the regulation of HBV infection and show that CDK4/6 inhibitors are beneficial for the viral life cycle. CDK4/6 promote the cell cycle G1/S transition by phosphorylating the retinoblastoma (Rb) protein, the gene product of a tumor-suppressor gene, and a central regulator of cell cycle progression^44^. CDKN2C interacts with CDK4/6 to block cell cycle G1 progression via Rb protein phosphorylation^45^. Here we find that *CDKN2C* overexpression in HBV-infected hepatocytes enhances replication in both NTCP-overexpressing hepatoma-derived cell line and in PHHs. Our results suggest an effect of CDKN2C on host cellular factors that are instrumental in HBV transcription. Indeed, CDKN2C overexpression is associated with an upregulation of transcription factors important for the HBV life cycle, such as *HNF4A*, *HLF*, and *PPARA* (Fig. 5i). Furthermore, we observed that overexpression of *CDKN2C* in HBV-producer cells resulted in a supernatant containing HBV particles that appeared to be consistently more efficient in infecting naive recipient cells (Fig. 6). It is conceivable that CDKN2C overexpression and the subsequent modulation of expression of HBV host-dependency factors (Fig. 5i) results in differences in virion assembly, which could explain this observation. Further experiments are needed to understand the functional role of CDKN2C for the formation of infectious particles. Overall, we identify *CDKN2C* as HBV host factor, acting through inhibition of CDK4/6 and prevention of G1 cell cycle progression.

The identification of *CDKN2C* as a host factor for HBV infection not only improves our understanding of the virus–host interactions but also contributes to the explanation of the poor permissivity of NTCP-overexpressing Huh7 cells to this virus (Fig. 1a). A detailed understanding of the molecular mechanisms underlying the varying susceptibility of currently available HBV infection models to HBV infection is crucial for the development of improved infectious cell culture models. The weak permissivity of Huh7-NTCP compared to HepG2-NTCP cells to HBV infection could be partially explained by lower *CDKN2C* expression levels (Fig. 3d, e). However, the lower cccDNA levels in Huh7-NTCP compared to HepG2-NTCP are not caused by the lack of *CDKN2C* as its overexpression does not affect cccDNA formation (Fig. 5d, e). Huh7-NTCP cells might therefore be useful to identify additional missing proviral host factors or restriction factors involved in cccDNA formation. Overall, a better understanding of virus–host interactions will facilitate the development of improved infectious model systems for drug discovery.

In patients, *CDKN2C* expression is accompanied with progression of HBV-associated fibrosis and is higher in HBV-associated cirrhotic and HCC tissues compared to tumor-adjacent tissues. In fact, *CDKN2C* is an etiology-independent marker of liver disease (Fig. 9) and part of a regulatory signature involved in liver regeneration^46^. This might explain the association of higher *CDKN2C* expression in HCC patients with lower long-term survival (Fig. 9). While the upregulation of CDK inhibitors in cancer cells may appear counterintuitive, our consistent observations of an association between *CDKN2C* expression and progressive liver disease and hepatocarcinogenesis in several independent cohorts (Fig. 9) are in line with a recent observation that the expression of the tumor-suppressor and *CDKN2C* effector Rb, which is regularly inactivated in human cancer, was inversely correlated with *CDKN2A*, another CDK4/6 inhibitor^47^.Given the positive correlation of CDKN2C expression and survival, it is likely that CDKN2C rather has procarcinogenic properties than a tumor-suppressive function in HCC (Fig. 9). It is also of interest to note that a recent study showed that pgRNA-positive HCCs were characterized by low levels of cell cycle and DNA repair markers and that pgRNA and cccDNA in tumors was correlated to the absence of tumorous microvascular invasion and to better patient survival^48^. However, while HBV viral load and *CDK2NC* expression showed a positive correlation trend in a well-defined small cohort shown in Supplementary Fig. 6, additional correlation analyses in other cohorts are needed to corroborate this finding. Collectively, it is likely that *CDKNC2* expression is regulated by multiple and possibly different mechanisms in the different phases of HBV infection and disease and even more so in the context of HCCs.

Interestingly, chemotherapeutic agents for cancer treatment cause immunosuppression and can lead to HBV reactivation in asymptomatic HBV carriers or patients with resolved HBV infection^49,50^. The list of chemotherapeutic agents associated with HBV reactivation is growing and includes anthracyclines, corticosteroids, platinum, vinca alkaloid, other small molecule agents, monoclonal antibodies, and immune modulators^49^. Therefore, several professional societies, including American Association for the Study of Liver Diseases and European Association for the Study of the Liver, recommend HBV screening in all cancer patients undergoing chemotherapy and immunization with HBV vaccine or prophylactic antiviral therapy^49^. However, awareness of this serious clinical problem is limited^49^ and needs to be considered in clinical trials for new treatments. CDK-inhibiting drugs are a novel class of cancer therapeutics and three CDK4/6 inhibitors, palbociclib, ribociclib (LEE011), and abemaciclib, are Food and Drug Administration and European Medicines Agency approved for the treatment of advanced ﻿hormone receptor-positive breast cancer and in clinical trials for other non-breast malignancies^51^. Palbociclib (PD-0332991) is now under evaluation for the treatment of different Rb protein-positive cancers^52–54^ and most importantly in clinical trials for the treatment of HCC^55,56^. Chronic HBV infection accounts for approximately 50% of cases of HCC worldwide^1^. In this study, we show that CDK4/6 inhibition by palbociclib enhances HBV replication by arresting cells in the G0/G1 phase of the cell cycle. Therefore, caution is warranted in the use of such agents for HCC treatment. Our findings have important clinical implications as they indicate that there might be a potential risk of HBV reactivation during therapy with a CDK4/6 inhibitor, such as palbociclib, currently evaluated for HCC treatment.

Taken together, our gain-of-function screening approach allowed the identification of key HBV host factors, such as CDKN2C, with clinical implications in patients. Our data pave the way for the development of more permissive infection systems for the study of virus–host interactions and the identification of previously undiscovered antiviral targets urgently needed for viral cure.

## Methods

### Human subjects

Human serum from patients with chronic HBV/HDV infection followed at the Strasbourg University Hospitals, Strasbourg, France was obtained with informed consent. PHHs were obtained from liver tissue from patients undergoing liver resection for liver metastasis at the Strasbourg University Hospitals with informed consent. Protocols were approved by the local Ethics Committee of the Strasbourg University Hospitals (CPP) and the Ministry of Higher Education and Research of France (DC-2016-2616). Human samples from HBV-infected patients followed at the Chang Gung Memorial Hospital (Taipei, Taiwan) were obtained with informed consent. Protocols were approved by the local Ethics Committee (Institutional Review Board 102-3825C).

### Cell lines and viruses

NTCP-overexpressing Huh-106 and HepG2-NTCP cell lines^5,57^ as well as human embryonic kidney 293T (HEK 293T)^58^ cell line have been described. PHHs were isolated and cultured as described^58^. Recombinant HDV production^5,57^ as well as purification of infectious HBV particles from the inducible human hepatoblastoma HepAD38 has been described^5,59,60^.

### Reagents and plasmids

DMSO, polybrene, and PEG 8000 (polyethylene glycol) were obtained from Sigma-Aldrich (Merck). DNA and RNA transfection at the indicated concentrations was performed using the CalPhos Mammalian Transfection Kit (Clonetech) and Lipofectamine RNAiMAX (Thermo Scientific) according to the manufacturers’ instructions, respectively. The ORF-encoding lentivirus constructs for validations were obtained from the RNAi Platform, Broad Institute of MIT and Harvard (Cambridge, MA, USA). Cell viability/proliferation was assessed using PrestoBlue Cell Viability Reagent (Invitrogen) according to the manufacturer’s instructions. Cell toxicity was assessed using LDH-Glo cytotoxicity assay (Promega) in the supernatant according to the manufacturer’s instructions. Palbociclib and LEE011 (Ribociclib) were obtained from Synkinase and Sellekchem, respectively.

### HBV binding

The binding of HBV virions at the cell surface was assessed as described^5^. In brief, ﻿cells were incubated with pretreated HBV in the presence of 4% PEG for 24 h at 16 °C. Unbound virions were removed by three washes with phosphate-buffered saline (PBS), and cells and bound virions were lysed. HBV total DNA was quantified by qPCR using a standard curve generated from known HBV genome copies.

### HBV and HDV infections

For HBV infection, NTCP-overexpressing cell lines and PHHs were infected by recombinant HBV in the presence of 4% of PEG-8000 (GEq 500 or 1000 per cell)^5,60^. After infection, Huh7-106 and HepG2-NTCP cells were washed and culture in PMM medium with 2% or 3.5% of DMSO, respectively for 10 days. HBV infection was assessed 10 dpi by IF using a mouse monoclonal antibody (Ab) targeting HBsAg (Bio-Techne, clone 1044/329, 1:100) and Alexa Fluor 647-labeled secondary Ab targeting mouse immunoglobulin G (IgG; Jackson Research, 1:200). Cell nuclei were stained with 4,6-diamidino-2-phenylindole. Fluorescent imaging was performed using an Axio Observer Z1 microscope (Carl Zeiss, Germany). Alternatively, cells were lysed and total RNA was extracted using the ReliaPrep RNA Miniprep Systems (Promega) and quantitative reverse transcriptase PCR (qRT-PCR) quantification of HBV pgRNA was assessed as described^5,60,61^. HBsAg and HBeAg secretion were quantified by chemiluminescence immunoassay (Autobio) following the manufacturer’s instructions. Southern blot detection of HBV cccDNA was performed using digoxigenin (DIG)-labeled (Roche) specific probes as described^62^. Total DNA from HBV-infected cells was extracted using the Hirt method as described^63^. Specific DIG-labeled probes for the detection of HBV and mitochondrial probes for the detection of HBV and mitochondrial DNAs were synthetized using the PCR DIG Probe Synthesis Kit (Roche) and the primers as described^60^. HBV total RNAs were detected by northern blot. Total RNA was purified using ReliaPrep RNA Miniprep Systems (Promega). Five µg of total RNA was subjected to electrophoresis through 2.2 M formaldehyde and 1% agarose gel and transferred to a nylon membrane positively charged (Roche). The membrane-bound RNA was hybridized to a ^32^P-labeled RNA probe specific for detection of HBV RNA of 1200–1944 bp of viral genome (3.5–2.1 kbp). Quantification of HBV DNA and RNA bands from blots was performed using Image Lab Version 5.2.1 (Bio-Rad). For HDV infection, NTCP-overexpressing cell lines were infected with recombinant HDV (GEq 100 per cell) as described^5,60^. HDV infection was assessed 7 days after infection by IF using an Ab targeting the hepatitis delta antigen (1:200) purified from serum of an HBV/HDV co-infected patient^64^ and AF647-labeled secondary Ab targeting human IgG (Jackson Research, 1:200) as described^5,65^.

### Genome-scale lentiviral expression library and gain-of function screen

hORFeome V8.1 library (Broad Institute of MIT and Harvard, Cambridge, MA, USA) containing a pool of 16,172 clonal ORFs (mapping 13,833 human genes) was cloned into a pLX_TRC317 vector. The establishment of the genome-scale ORFeome library has been described^18^. Thirty million Huh-106 cells were transduced with the lentiviral ORFeome library in duplicate in the presence of polybrene (4 µg/mL). To avoid a cumulative effect of multiple ORFs, the lentivirus volume was optimized to obtain 30% of transduced cells. Cells were then selected with puromycin (0.9 µg/mL) for 3 days. After amplification, transduced cells were infected with recombinant HBV at an MOI of 1000 GEq/cell or mock-infected. At 10 dpi, cells were stained for HBsAg expression and sorted by flow cytometry.

### Gene expression analysis in HBV-infected Huh-106 after ORFeome transduction

HBV-infected cells were fixed in 100% methanol for at least 20 min at −20 °C. Cells were then blocked and permeabilized using PBS–0.5% bovine serum albumin (BSA) and 0.05% saponin for 1 h at room temperature (RT). Cells were stained using an AF647-conjugated mouse monoclonal anti-HBsAg Ab (Bio-Techne, clone 1044/329) and resuspended in 0.5% BSA. HBsAg-positive cells were sorted by FACS (BD FACSAria Flow Cytometer). Twenty million cells were taken from HBV-infected sample as pre-sort control and total genomic DNA (gDNA) was extracted from cell pellets using Qiagen kits according to the manufacturer’s protocol (Qiagen). In addition, gDNA was extracted from 20 million HBV-positive sorted cells from two biological replicates, named HBV sorted. Extracted DNA was used as a template for PCR to amplify the barcode sequences that accompany every ORF in the library. The unique barcode associated with each ORF construct was determined by Sanger sequencing in an arrayed collection of all the ORF constructs prior to pooling. PCR and sequencing were performed as previously described^66,67^. The details of the PCR primers and conditions can be found here: https://portals.broadinstitute.org/gpp/public/resources/protocols. Samples were sequenced on a HiSeq2000 (Illumina). The resulting reads were matched to their barcodes and their associated ORFs using PoolQ (see https://portals.broadinstitute.org/gpp/public/resources/protocols for more information on PoolQ). For analysis, the read counts were normalized to reads-per-million and then log2 transformed. Log2FC of each ORF was determined relative to the initial time point for each biological replicate. Ninety hits with Log2FC values above the threshold set at 1.5 were selected as candidates.

### Flow cytometry

For further flow cytometric analysis of HBV-infected cells, cells were fixed in 100% methanol for at least 20 min at −20 °C. Cells were then blocked and permeabilized using PBS–1% fetal bovine serum and 0.05% saponin for 30 min at RT. HBsAg was stained using a mouse monoclonal anti-HBsAg Ab (Bio-Techne, clone 1044/329, 1:1000) for 30 min at 4 °C and then with an AF647-labeled secondary Ab targeting mouse IgG (Jackson Research, 1:1000) for 30 min at 4 °C. For flow cytometric analysis of DNA content, cells were fixed in ice-cold 75% ethanol in water for 30 min at 4 °C. Cells were washed and resuspended and incubated in PBS 50 µg/mL propidium iodide (Invitrogen) and 50 µg/mL Ribonuclease A (Sigma-Aldrich, Merck) for 30 min at RT. Cells were subsequently washed and resuspended in PBS–5 µM EDTA prior to sorting through a CytoFLEX flow cytometer system (Beckman Coulter). The gating strategy is presented in Supplementary Fig. 7.

### Candidate selection from the primary screen

The impact of gene overexpression on HBV infection was defined by a specific enrichment in cDNA sequences in HBV-positive sorted cells compared to the pre-sort population. For hit selection, a functional threshold of Log2FC = 1.5 compared to pre-sorted cells was applied, leading to a total of 90 candidates (Supplementary Table 1, Fig. 2c, d). As multiple ORF sequences for one given gene are sometimes present in the library, individual sequences were analyzed. Candidate genes with multiple associated ORFs were selected only if clones presented significant differences in their sequences (truncations in Cter or Nter of the proteins) or if at least two identical ORFs exhibited a Log2FC > 1. Candidate gene expression in the liver was then assessed through the Human Protein Atlas (available from www.proteinatlas.org)^68^. Candidates with liver expression <0.1 transcript per million were removed from the analysis, leading to a final selection of 47 candidates (Supplementary Table 1). Forty-seven ORF-containing lentiviruses were then obtained for individual validations, 35 of which met internal quality control based on lentiviral titration. In addition, lentiviruses encoding *GFP*, *KRT80*, and *CPA1* cDNA sequences were obtained as negative controls from the primary screen.

### Hit validation in Huh-106 cells and PHHs

Individual ORFs were expressed from pLX-Blast-V5 (lentiviral) expression plasmids. Lentivirus particles were produced in HEK 293T cells by cotransfection of plasmids expressing the human immunodeficiency virus gap-pol, the vesicular stomatitis virus glycoprotein, and the pLX-Blast-V5-ORF plasmids in the ratio of 10:3:10, using the CalPhos Mammalian Transfection Kit as described^58^. Three days after transfection, supernatants were collected, pooled, and clarified using 0.45-µm pore filters. Huh-106 were individually transduced with the 38 ORF-expressing lentivirus constructions and selected with 6 µg/mL of blasticidin 48 h prior to HBV infection. HBV infection was assessed after 10 days by quantification of HBeAg and HBsAg expression in the supernatant of infected cells as described above. For further validations, PHH and Huh-106 were transduced with individual ORF-containing lentivirus prior to HBV infection. Infection was assessed after 10 days by Southern blot detection of HBV DNA, northern blot and qRT-PCR detection of HBV RNAs, immunodetection of HBsAg, and quantification of HBeAg as described above.

### CDKN2C HepG2-NTCP KO generation

To generate clonal HepG2-NTCP CDKN2C KOs, the following primers corresponding to guide RNAs targeting CDKN2C exons were cloned into the Zhang laboratory-generated Cas9 expressing pX458 plasmid (Addgene plasmid #48138): guide 1; Fw: 5’-CACCGACACCGCCTGTGATTTGGCC-3’, Re: 5’-AAACGGCCAAATCACAGGCGGTGTC-3’. guide 2; Fw: 5’-CACCGCACAGGCGGTGTCCCCCTTA-3’, Re: 5’-AAACTAAGGGGGACACCGCCTGTGC-3’. pX458 plasmids encoding guide RNAs against CDKN2C were transfected into HepG2-NTCP cells using Lipofectamine 3000 (Life Technologies) according to the manufacture’s guidelines. Transfected cells were single cell sorted based on +GFP expression into 96-well plates using the SONY SH800S cell sorter. Individual clones were expanded, and four clonal cell lines were eventually selected for further characterization.

### RNAi loss-of-function studies

ON-TARGETplus siRNA pools (Dharmacon) targeting the transcripts of *CDKN2C* and *SLC10A1* (NTCP) were reverse-transfected into HepG2-NTCP cells with Lipofectamine RNAiMAX (Invitrogen) as described. RNA was purified from cells harvested 2 days after transfection, and gene expression was analyzed by qRT-PCR. For silencing of *CDKN2C* expression in PHHs, PHHs were transduced with lentiviral vectors containing *CDKN2C*-targeting shRNA (target sequence: GATGTTAACATCGAGGATAAT) or a scrambled shRNA control (target sequence: CCTAAGGTTAAGTCGCCCTCG) obtained from VectorBuilder. RNA was purified from PHHs harvested 3 days after transduction, and gene expression was analyzed by qRT-PCR.

### Comparative analysis of gene expression in Huh-106 and HepG2-NTCP cells

Huh-106 and HepG2-NTCP cells were lysed and total RNA from three biological replicates per cell line was then extracted as described above. Microarray analysis of gene expression in both cell lines was performed at the IGBMC GenomEast platform (Illkirch, France). Biotinylated single-strand cDNA targets were prepared from 200 ng of total RNA using the Ambion WT Expression Kit (Cat # 4411974) and the Affymetrix GeneChip® WT Terminal Labeling Kit (Cat # 900671) according to Affymetrix recommendations. Following fragmentation and end-labeling, 3 μg of cDNAs were hybridized for 16 h at 45 °C on GeneChip® Human Gene 2.0 ST arrays (Affymetrix) interrogating >400,000 RefSeq transcripts and ~11,000 long non-coding RNAs. The chips were washed and stained in the GeneChip® Fluidics Station 450 (Affymetrix) and scanned with the GeneChip® Scanner 3000 7G (Affymetrix) at a resolution of 0.7 µm. Raw data (CEL Intensity files) were extracted from the scanned images using the Affymetrix GeneChip® Command Console (AGCC) version 4.1.2. CEL files were further processed with the Affymetrix Expression Console software version 1.4.1 to calculate probe set signal intensities using Robust Multi-array Average algorithms with default settings. Modulated molecular pathways were determined by using gene set enrichment analysis^69^. Individual differential gene expression of the selected candidates was evaluated through the *Z* score transformation. The dataset is publicly available in the NCBI Gene Expression Omnibus database (accession number GSE132638).

### Analysis of gene expression using qRT-PCR

RNA was extracted as described above, and gene expression was assessed by qRT-PCR as described^60^. Gene expression was normalized to *GADPH* expression. Primers and TaqMan® probes for quantification of *GAPDH*, *CDKN2C*, and *SLC10A1* mRNA expression were obtained from ThermoFisher (TaqMan® Gene Expression Assays). Gene expression was quantified using iTaq Universal Probes Supermix (Bio-Rad). Primers for quantification of *HNF4A* (Fw: 5’-ACATTCGGCAAGAAGATT-3’; Re: ACTTGGCCCACTCAACGAG-3’), *HLF* (Fw: 5’CACCACGAAGACGATTTAG-3’; Re: 5’-CAAAAACTCCTCCAGGTCCA-3’), *PPARA* (Fw: 5’-GAGGGTCTCCACTGACGTG-3’; Re: 5’-ACACTGTGTATGGCTGAGAAG-3’), and *GAPDH* expression (Fw: 5’-GTCTCCTCTGACTTCAACAGCG-3’; Re: 5’-ACCACCCTGTTGCTGTAGCCAA-3’) were obtained from Sigma-Aldrich (Merck). Gene expression was quantified using iTaq Universal SYBR Green Supermix (Bio-Rad).

### Protein expression

The expression of CDKN2C and *β*-tubulin was assessed by western blot as described^5^ using a monoclonal rabbit anti-CDKN2C Ab (anti-p18 INK4c, ab192239, Abcam, 1:1000), a rabbit polyclonal anti-*β*-tubulin Ab (GTX101279, Gentex, 1:3000), and a rabbit polyclonal anti-glyceraldehyde 3-phosphate dehydrogenase (anti-GAPDH; ab9485, Abcam, 1:2500), respectively. Peroxidase-AffiniPure Goat Anti-Rabbit IgG (H+L) (Jackson Research 111-035-144, 1:10,000) was used as a secondary Ab. Protein expression was assessed using the ChemiDoc™ Imaging System (BioRad).

### Analysis of nascent HBV RNA synthesis

Run-on assays were performed using the Click-iT™ Nascent RNA Capture Kit from ThermoFisher Scientific according to the manufacturer’s instructions. HBV total and nascent RNA expression was assessed from HBV-infected Huh-106 cells overexpressing either *GFP* or *CDKN2C* by qRT-PCR 4 days after virus inoculation with 2 h of ethynyl uridine (EU) labeling. Actinomycin D (ActD, Sigma-Aldrich, Merck) was used as a negative control. Cells were pretreated with ActD at 10 mg/mL for 20 min prior to EU labeling in the presence of ActD. Specific primers and TaqMan® probes for total HBV RNAs (Pa03453406_s1) were purchased from Life Technologies. HBV RNA levels were normalized to *GUSB* expression using primers and TaqMan® probes from Life Technologies (Hs99999908_m1).

### Analysis of *CDKN2C* expression in patients

For the analysis of *CDKN2C* mRNA expression in patients, *CDKN2C* mRNA expression was assessed in control healthy patients (*n* = 6), HBV-infected patients with no detectable HBV DNA (*n* = 32), and HBV-infected patients with detectable HBV DNA (*n* = 90) from GSE83148^70^. Similarly, CDKN2C mRNA expression was assessed in HBV patients at different stages of virus infection, including immune tolerant phase (*n* = 22), immune clearance phase (*n* = 50), and inactive carrier phase (*n* = 11) from GSE65359. Alternatively, total RNA was extracted from liver tissue of nine HBV-infected patients by using the High Pure RNA Paraffin Kit (Roche) according to the manufacturer’s instruction, and gene expression analysis was performed by RNA-seq as previously reported^71^. To analyze the correlation between *CDKN2C* expression and the progression of liver disease in HBV-infected patients, *CDKN2C* mRNA expression was assessed in HBV-related liver fibrosis patients of different stages from GSE84044^72^ (*n* = 37 score 0, *n* = 33 score 1, *n* = 34 score 2, *n* = 15 score 3). Finally, *CDKN2C* expression in HBV-induced HCC patients was assessed from GSE65485^73^ (*n* = 50 tumor tissue, *n* = 5 non-tumor tissue) and from GSE14520^74^ (*n* = 221 tumor tissue, *n* = 199 non-tumor tissue). *CDKN2C* mRNA expression is shown as signal intensity values. For survival analysis, liver expression level of *CDKN2C* and survival data were derived from The Cancer Genome Atlas (TCGA; https://www.cancer.gov/about-nci/organization/ccg/research/structural-genomics/tcga) TCGA-LIHC database^75^. To analyze *CDKN2C* expression in liver tissue of patients with chronic liver disease, fragments per kilobase of transcript per million mapped read values and clinical data were retrieved from TCGA. This dataset includes samples from HCV-infected patients (34 tumor samples including 5 paired tumor/non-tumor samples), HBV-infected patients (76 tumor samples including 7 paired tumor/non-tumor samples), patients with ALD (72 tumor samples including 8 paired tumor/non-tumor samples), and patients with NAFLD (11 tumor samples including 2 paired tumor/non-tumor samples).

### Statistics and reproducibility

Individual experiments were reproduced three times in an independent manner with similar results except otherwise stated. The precise number (*n*) of biologically independent samples used to derive statistics is indicated in the figure legends. For in vitro experiments, statistical analyses were performed using a two-tailed Mann–Whitney *U* test; *p* < 0.05 (*), *p* < 0.01 (**), and *p* < 0.001 (***) were considered statistically significant. Significant *p* values are indicated by asterisks in the individual figures and figure legends. The exact *p* values are provided in the Source Data file. For *n* < 10, the corresponding data points are presented with the bar charts. For microarray analyses, two-tailed unpaired Student’s *t* test was performed by comparing the values from three biological replicates per cell line. *p* < 0.01 was considered statistically significant. For clinical data, Mann–Whitney *U* test was used when comparing two groups (Fig. 9d, e). For multiple group comparison (Fig. 9b, c), Kruskal–Wallis *H* test adjusted for multiple comparisons was used. Correlation between *CDKN2C* expression and HBV viral load in patients was assessed using Spearman’s rank correlation coefficient (Spearman’s rho). Survival functions depending on *CDKN2C* expression were obtained using the Kaplan–Meier estimator. *p* value was calculated using log-rank test for comparisons of Kaplan–Meier survival. *p* < 0.01 was considered statistically significant. Representative graphs and pictures presented in Figs. 1a, c, e; 4c; 5a, d, f; and 7f are representative of three independent experiments with similar results. Representative graph presented in Fig. 3e is representative of two independent experiments with similar results. Graphs were designed using GraphPad PRISM 6 for Windows and Microsoft Excel for Microsoft Office 365 ProPlus (version 1911).

### Reporting summary

Further information on research design is available in the Nature Research Reporting Summary linked to this article.

## Supplementary information

Supplementary Information

Reporting Summary

## Data Availability

The dataset generated in this study, including the results from the gain-of-function primary screen, are available within Supplementary information. Full immunoblots are provided in Supplementary Fig. 8. The microarray dataset is publicly available in the NCBI Gene Expression Omnibus database (accession number GSE132638). The source data underlying Figs. 1, 2, 3, 4, 5, 6, 7, and 9 and Supplementary Figs. 1, 4, 5, and 6 are provided as a Source Data file. The details of the box plots presented in Fig. 9b–e and Supplementary Fig. 6b are presented in Supplementary Table 2 and Supplementary Table 3. The rest of the data is available through the corresponding authors upon reasonable request. The following public databases were used in the study: GSE83148; GSE65359; GSE84044; GSE65485; GSE14520; TCGA-LIHC [https://www.cancer.gov/about-nci/organization/ccg/research/structural-genomics/tcga]. The following public protocol was used: PoolQ [https://portals.broadinstitute.org/gpp/public/resources/protocols].

## Source Data

Source Data
