# Supplementary material for: A genome-wide gain-of-function screen identifies CDKN2C as a HBV host factor
Source: Nat Commun. 2020 Jun 1;11:2707. doi: 10.1038/s41467-020-16517-w (PMC7264273; doi:10.1038/s41467-020-16517-w)
Supplement: Supplementary file 1 — Supplementary Information [file 41467_2020_16517_MOESM1_ESM.pdf]

A genome-wide gain-of-function screen identifies CDKN2C as a HBV host factor

Carla Eller[11], Laura Heydmann[11], Che C. Colpitts, Houssein El Saghire, Federica Piccioni, Frank Jühling, Karim Majzoub, Caroline Pons, Charlotte Bach, Julie Lucifora, Joachim Lupberger, Michael Nassal, Glenn S. Cowley, Naoto Fujiwara, Sen-Yung Hsieh, Yujin Hoshida, Emmanuele Felli, Patrick Pessaux, Camille Sureau, Catherine Schuster, David E. Root, Eloi R. Verrier[12], Thomas F. Baumert[12]

[11]These authors contributed equally. [12]These authors jointly supervised this work.

SUPPLEMENTARY INFORMATION

SUPPLEMENTARY FIGURES

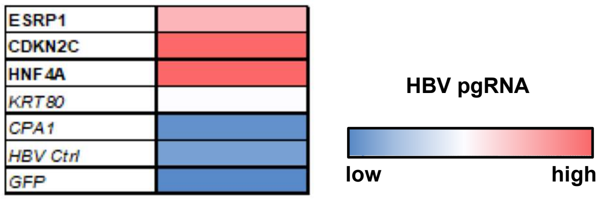

Supplementary Figure 1. Heatmap of candidate validation by quantification of pgRNA (related to Figure 3a). Huh-106 cells were transduced with the indicated ORF and infected with HBV. HBV infection was assessed at 10 dpi by qRT-PCR quantification of pgRNA. Results are expressed as means relative pgRNA expression from 2 independent experiments (n=4). Source data are provided as a Source Data file.

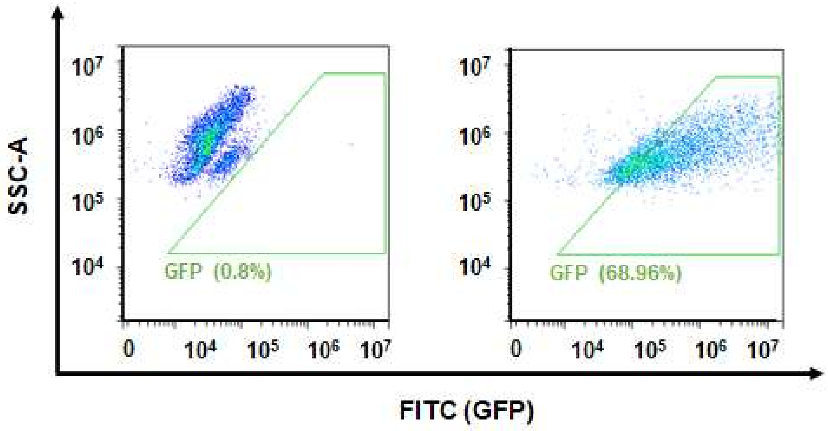

Supplementary Figure 2. GFP control vector (related to Figure 5b). Expression of GFP in Huh-106 transduced with lentivirus for GFP overexpression (GFP) or non-transduced (NT). Quantification of GFP-expressing cell population by flow cytometric analysis in HBV-infected cells 10 dpi.

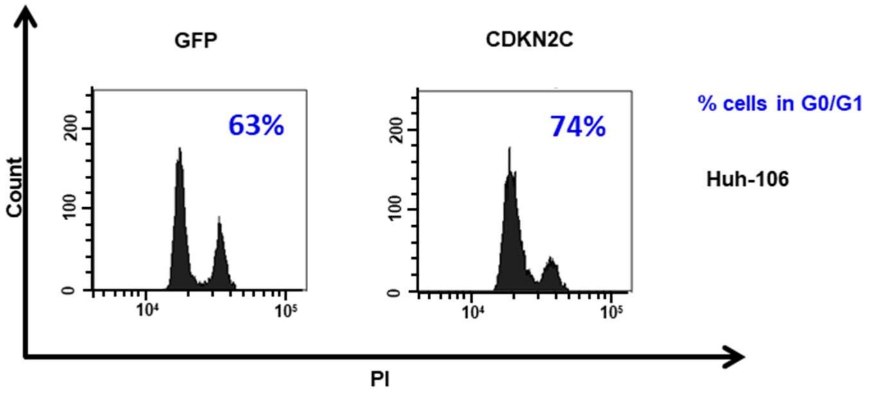

Supplementary Figure 3. Effect of *CDKN2C* overexpression on cell cycle (related to Figure 7). Overexpression of GFP or CDKN2C in Huh-106 cells and cultivation in Williams Culture and 2% DMSO. Analysis of cell cycle using propidium iodide and flow cytometry after 3 days. One representative experiment out of 3 is shown (n=4).

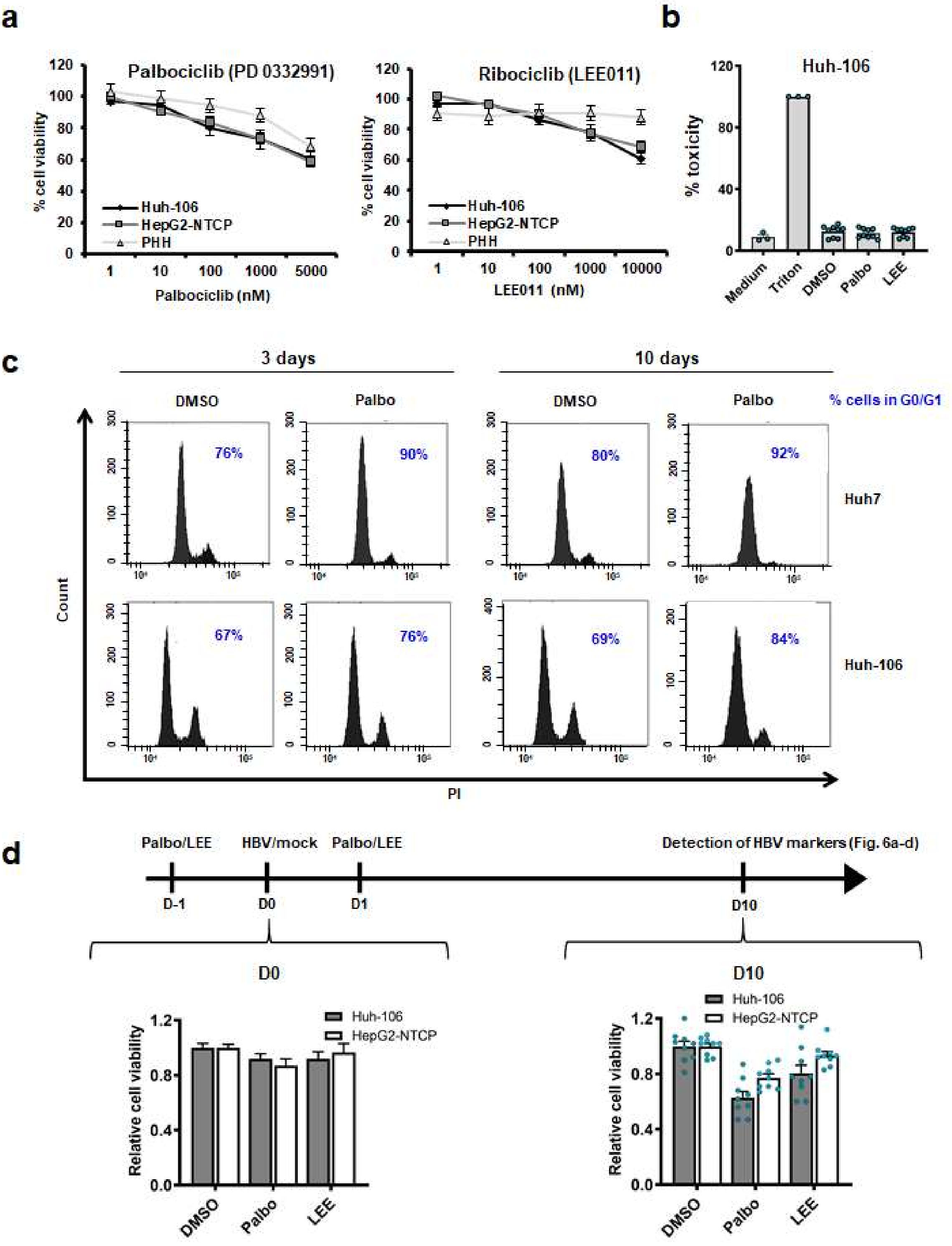

Supplementary Figure 4. Effect of treatment with CDK4/6 inhibitors Palbociclib (Palbo) and LEE011 on cell viability, cytotoxicity and cell cycle (related to Figure 7). **a** Cell Viability. Cell viability was assessed by the PrestoBlue assay in Huh-106 cells, HepG2-NTCP cells and PHH from 3 different donors treated with different concentrations of Palbociclib (Palbo) or LEE011 for 3 days in 0.1% DMSO. Results are expressed as means +/- SEM % cell viability compared to 0.1% DMSO treated cells (set to 100%) from 3 independent experiments (n=10, n=12 for PHH). **b** Toxicity in Huh-106 cells. Direct cytotoxicity was measured using the LDH-Glo cytotoxicity assay (Promega). Cells were treated for three days with the compounds at a concentration of 100 nM or with 0.1% DMSO as a negative control. Results are expressed as means +/- SEM % cytotoxicity compared to 10% Triton (Triton) treated cells (set to 100%) from 3 independent experiments (n=9). Medium: cell culture medium control (basal detection). **c** Effect on cell cycle. Huh7 and Huh-106 cells were treated with DMSO as negative control or with 100 nM Palbociclib (Palbo) in Williams Culture and 2% DMSO. Analysis of cell cycle using propidium iodide and flow cytometry was performed after 3 days and 10 days. One representative experiment is shown (n=4). **d** Cell viability (PrestoBlue) in Huh-106 cells, HepG2-NTCP cells treated with 100 nM Palbociclib (Palbo) or 100 nM LEE011 (LEE) for 1 day (D0) or for 1 day before HBV infection and 9 days after removal of HBV inoculum (D10). Results are expressed as means +/- SEM % cell viability compared to DMSO-treated cells (DMSO – set 1) from 3 independent experiments (n=12) for D0 or as means +/- SEM relative cell viability compared to untreated HBV-infected cells (DMSO - set to 1) from 3 independent experiments (n=9). Source data are provided as a Source Data file.

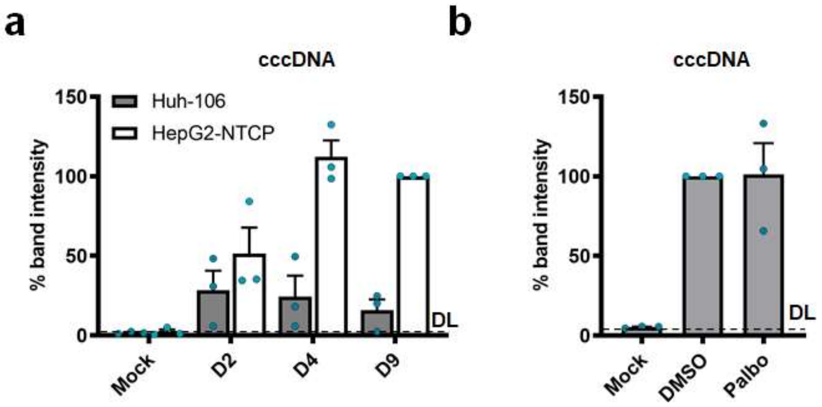

Supplementary Figure 5. Quantification of Southern Blot cccDNA band using Image Lab Version 5.2.1 (related to (a) Figure 1e and (b) Figure 7f). a Quantification of cccDNA bands in Mock or HBV infected Huh-106 and HepG2-NTCP cells 2 (D2), 4 (D4) or 9 (Mock, D9) days post HBV infection. Results are expressed as means +/- SEM % band intensity compared to HBV-infected HepG2-NTCP D9 (set to 100%) from 3 independent experiments. Dashed line indicates the detection limit (DL). Related to Figure 1e. **b** Quantification of cccDNA bands in Mock or HBV infected Huh-106 cells treated with DMSO or 100 nM Palbociclib 4dpi. Results are expressed as means +/- SEM % band intensity compared to DMSO (set to 100%) from 3 independent experiments. Dashed line indicates the detection limit (DL). Related to Figure 7f. Source data are provided as a Source Data file.

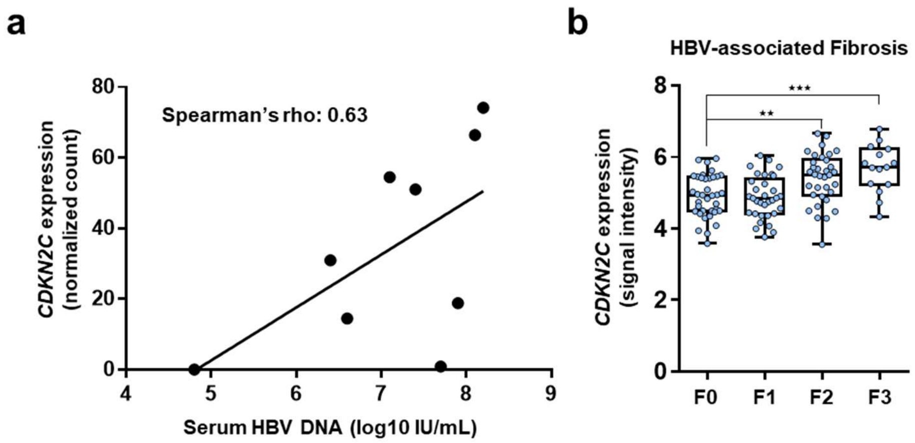

Supplementary Figure 6. CDKN2C expression is associated with HBV infection and survival in patients (related to Figure 9). a Correlation between HBV DNA and *CDKN2C* expression in 9 HBV-infected patients. Serum HBV-DNA levels (log10 IU/mL) and liver tissue CDKN2C expressions showed a trend toward a positive correlation (Spearman's rho = 0.63, p = 0.076). **b** CDKN2C expression in patients with HBV-associated liver fibrosis at different stages of fibrosis (F0: n=37; F1: n=33; F2: n=34; F3: n=15). ** p < 0.01; *** p < 0.001 (Kruskal–Wallis H test adjusted for multiple comparisons). Source data are provided as a Source Data file.

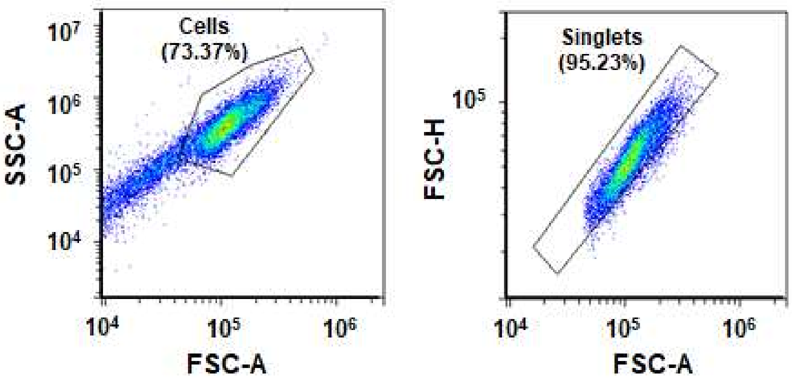

Supplementary Figure 7 (related to Figures 2 and 7). Flow cytometry gating strategy using Huh-106 cells. The gating was performed using an FCS/SSC dot plot. The gain was adjusted when all cell populations were visible on the plot. The living cell population was gated to perform a "singlets" plot through FSC-H and FSC-A parameters. Histogram- and dot plots were obtained from the "singlets" gate using Count/APC-A or SSC-A/APC parameters.

Supplementary Figure 8. Full immunoblots

a

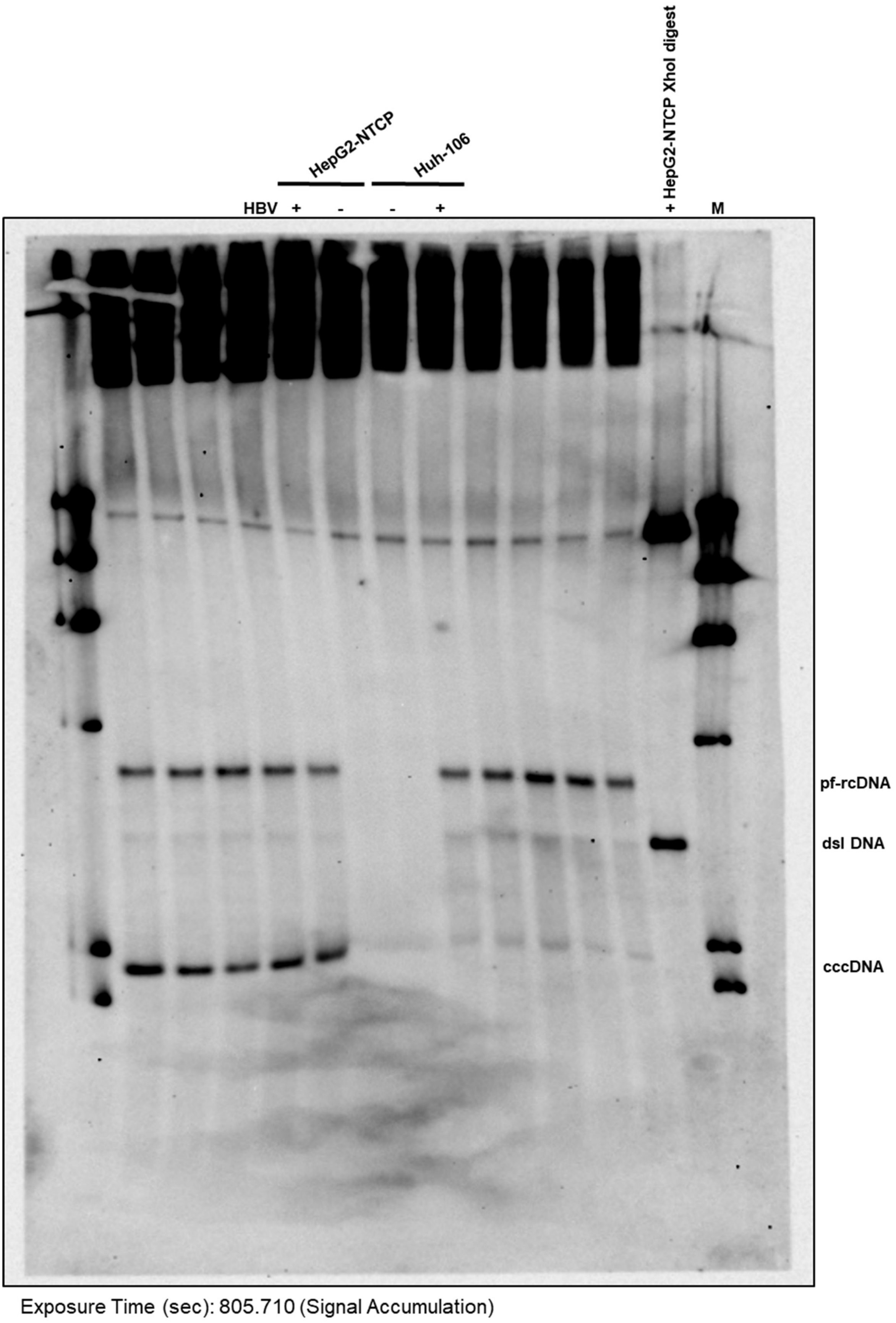

(a) related to Figure 1c, d

b

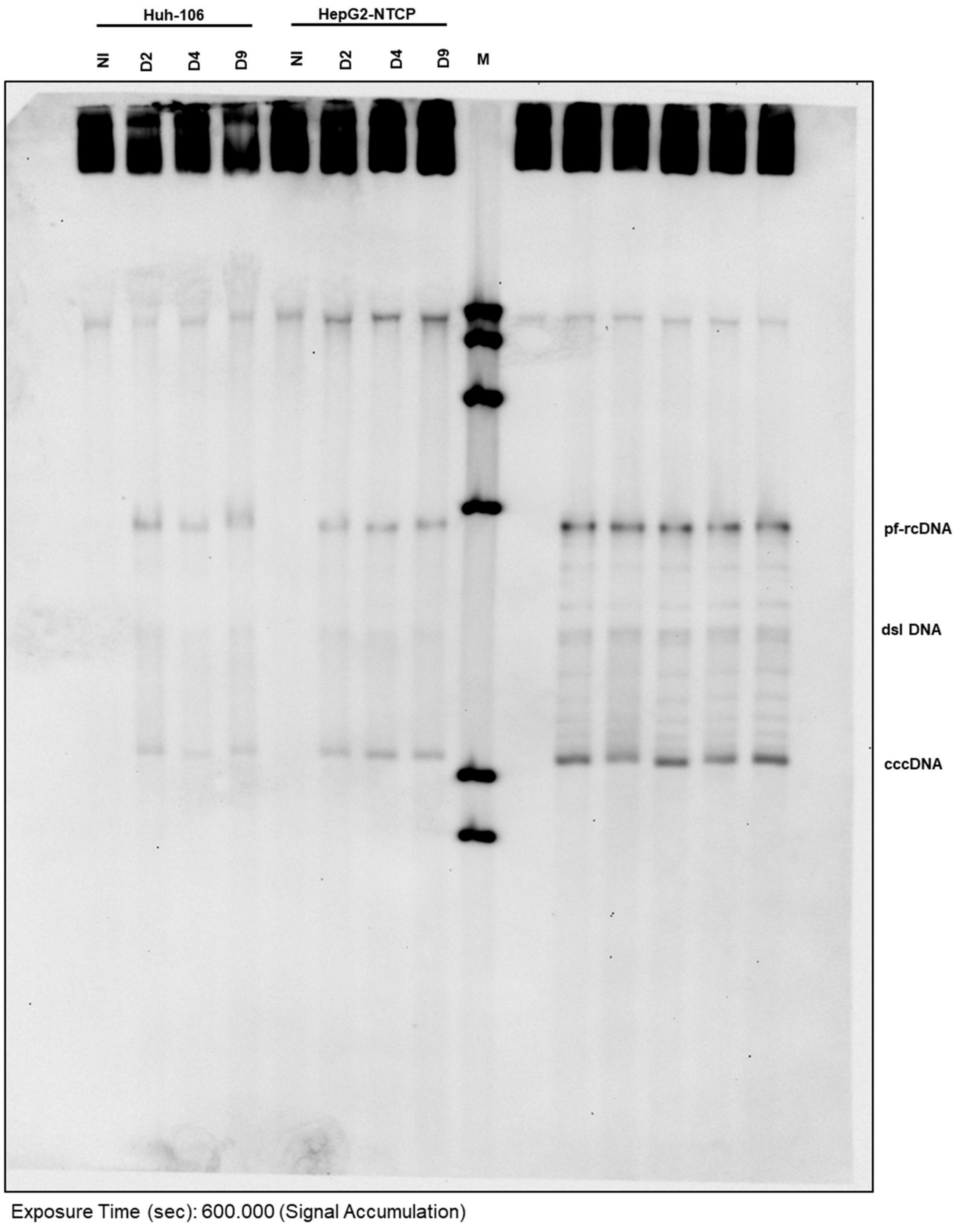

(b) related to Figure 1e, S5a

c

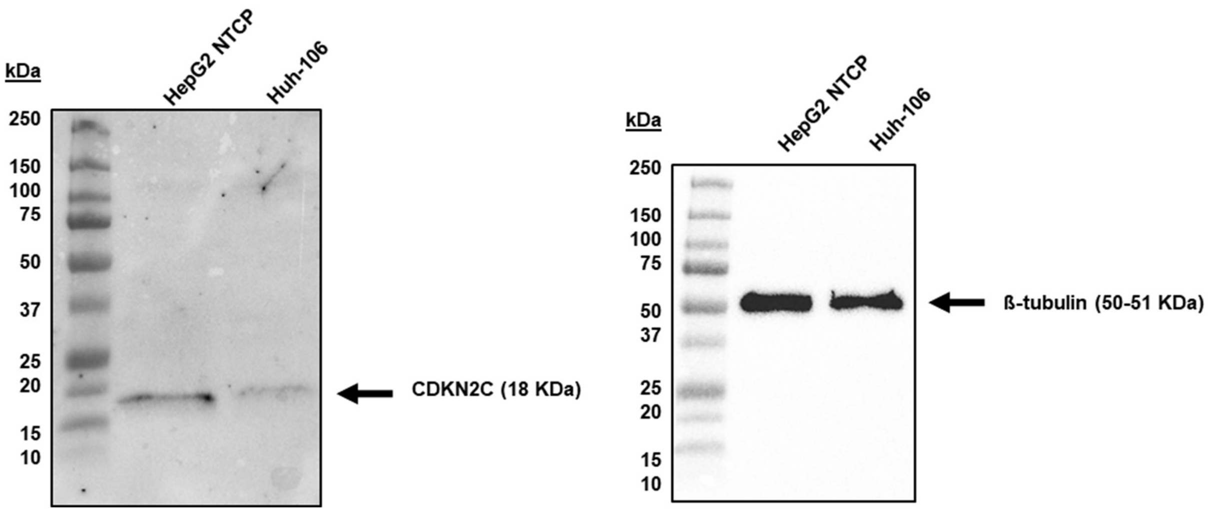

(c) related to Figure 3e

d

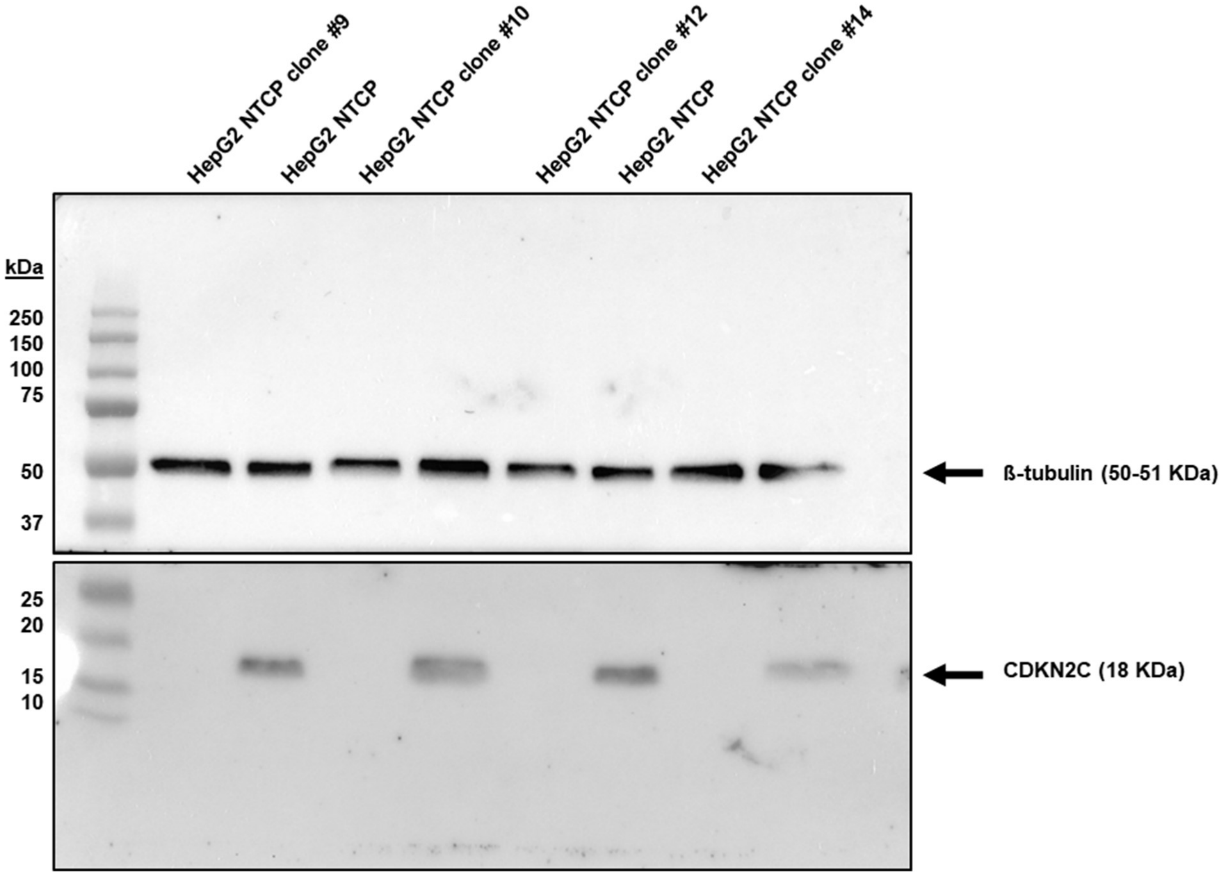

(d) related to Figure 4d

e

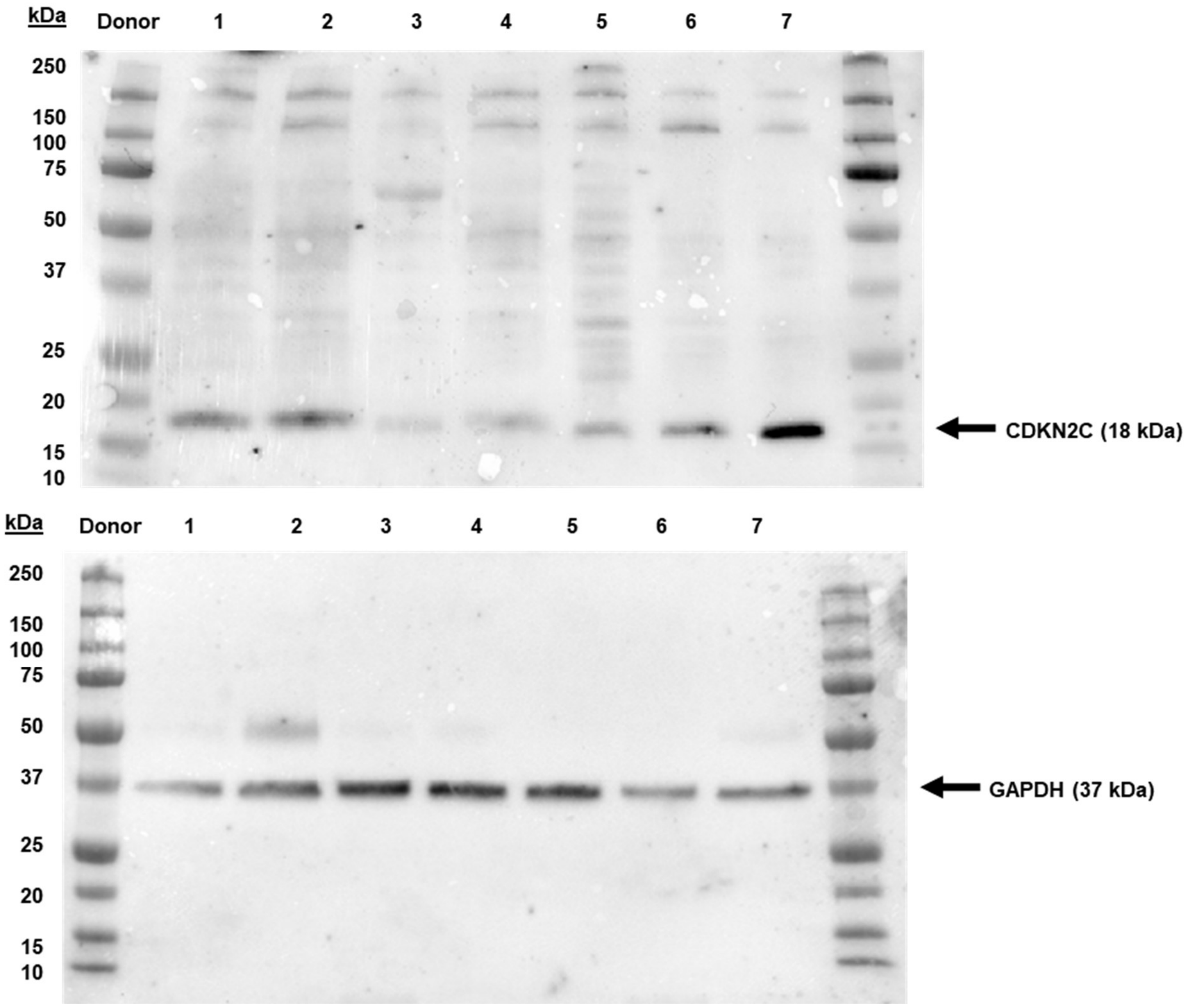

(e) related to Figure 4f

f

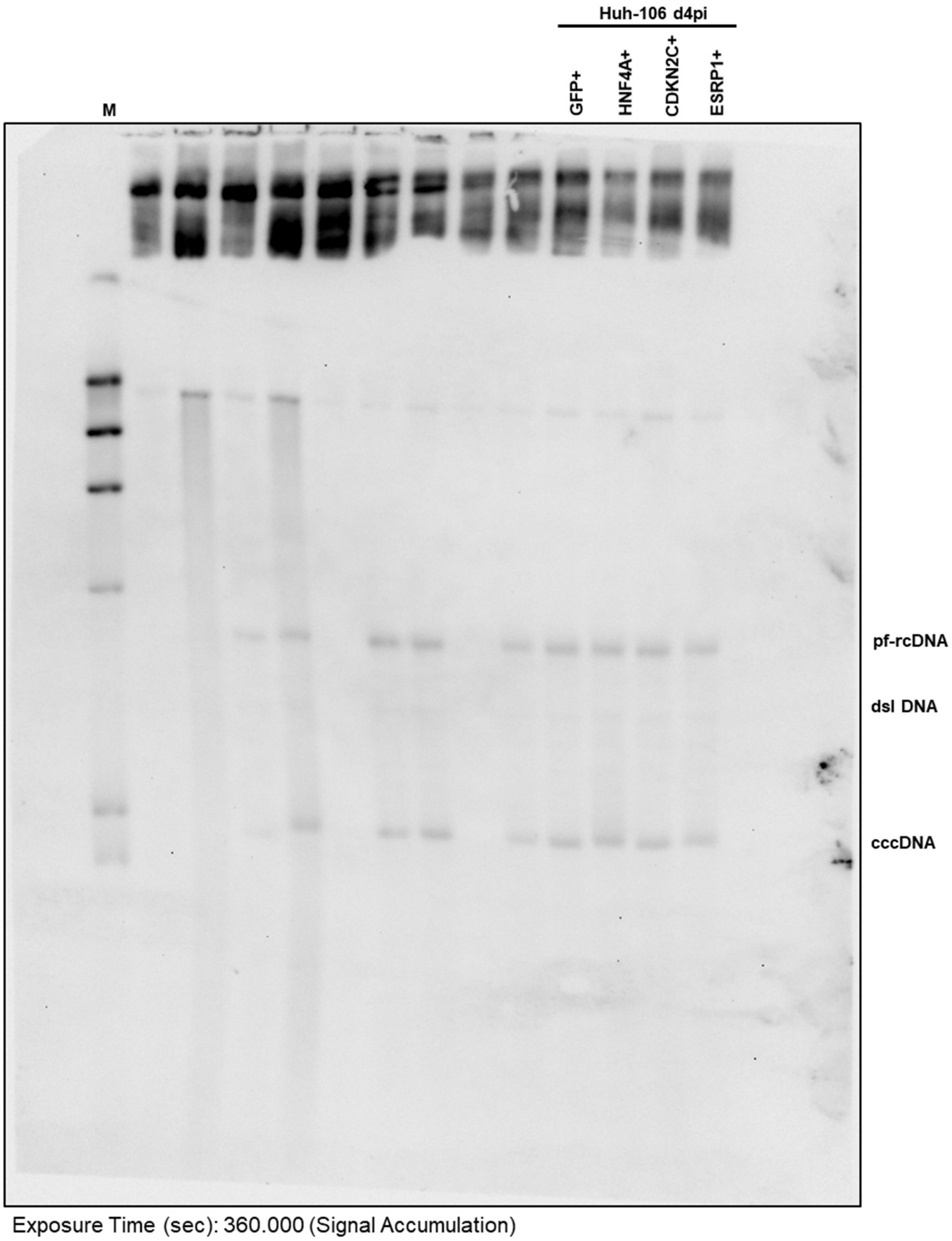

(f) related to Figure 5d, e

g

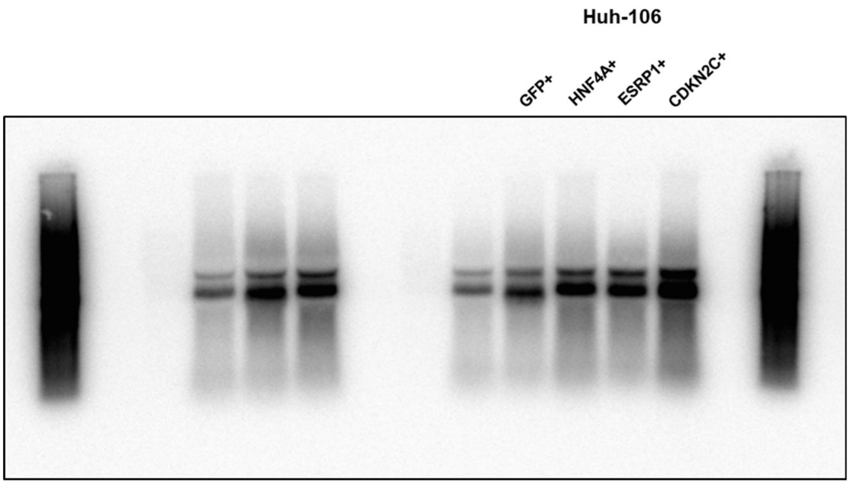

(g) related to Figure 5f, g

h

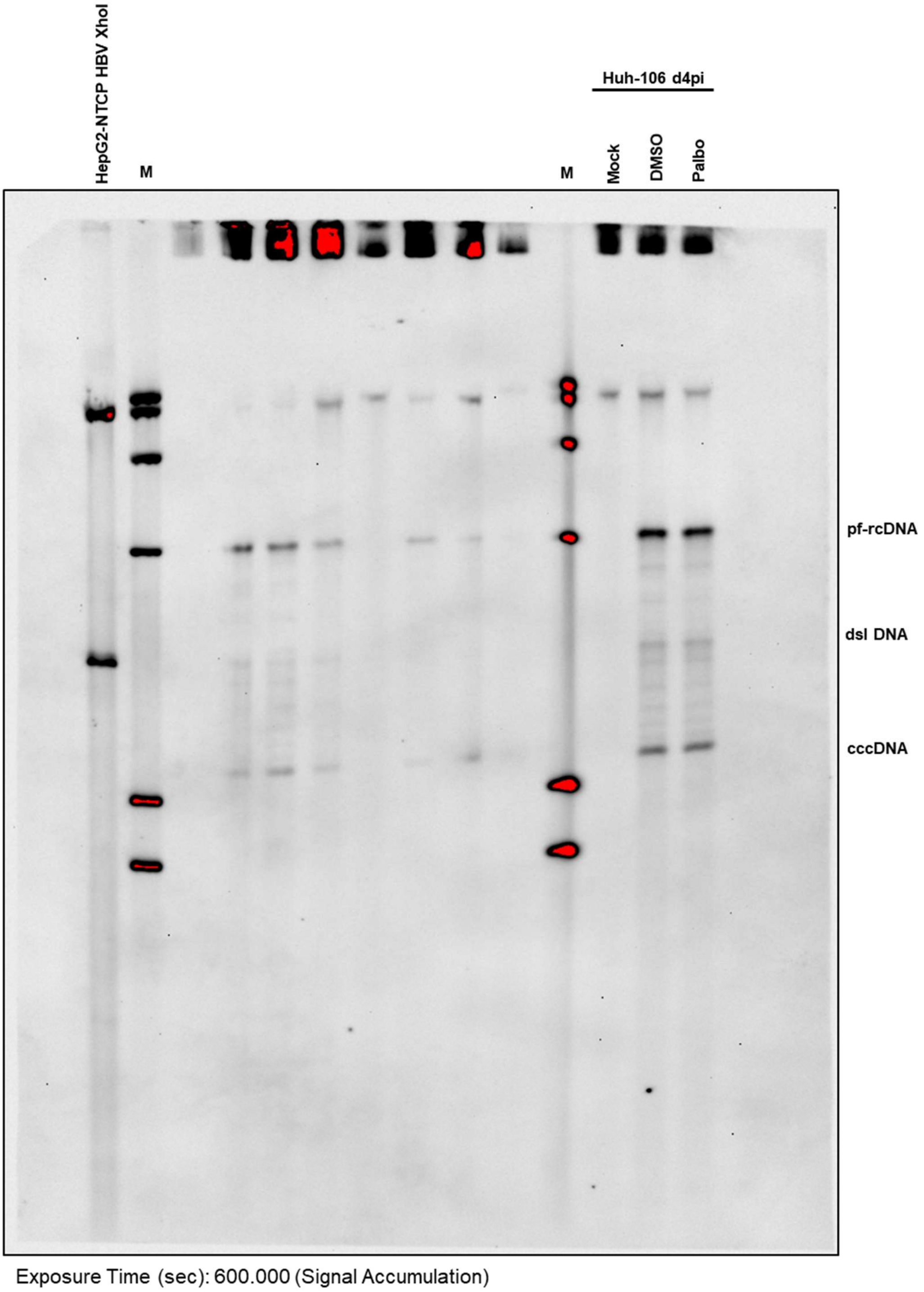

(h) related to Figure 7f, S5b

SUPPLEMENTARY TABLES

Supplementary Table 1. Candidates from the primary screen

Gene Symbol	AVERAGE LFC HBV presort	Clone filter	Expression in the liver (HPA - TPM)
HIST1H4B	1.57	X	56.8 (FANTOM5)*
ASGR1	1.60	X	236.1
SDC1	1.59	X	144.7
TOB1	1.56	X	58.5
HLA-DRB3	1.90	X	49.5
USO1	1.93	X	40.1
CLEC1B	2.20	X	33.7
DEK	1.51	X	31.6
FGFR1OP	1.68	X	18.7
MAPK1IP1L	2.24	X	14
U2AF1	1.77	X	13.9
HCCS	2.37	X	13.6
ASMTL	1.55	X	12.9
TRIM24	1.69	X	11.9
MFSD1	1.50	X	11.5
NOTCH2	2.19	X	9.9
NGEF	2.25	X	9
TMEM38B	2.47	X	7.9
KIAA0232	2.69	X	7.8
LAMC1	1.64	X	7.1
HNF4A	1.52	X	6.9
ZNF326	1.52	X	6.9
PPP2R5D	2.49	X	6.7
KLHL15	1.51	X	6.6
VPS45	1.75	X	6.2
GRK5	1.63	X	6
CREB1	1.52	X	5.7
WWP2	1.90	X	5.6
ENTPD4	1.67	X	5.1
TCF3	1.59	X	4.6
PRKD2	2.06	X	3.7
ABHD8	1.58	X	3.2
CDKN2C	1.63	X	2.7
TOMM40L	1.57	X	2.6
TTLL3	2.23	X	2.6
SPATA24	1.68	X	2.5
ZNF37A	1.69	X	2.5
ZNF354A	2.07	X	1.7
RUFY2	2.24	X	1.2
ARPP21	2.18	X	1.1
KDF1	2.00	X	1.1
SEMA4A	1.67	X	1.1
LIPE	1.51	X	0.4
ESRP1	1.69	X	0.2
GPR123	2.04	X	0.1
GPR27	1.69	X	0.1
SLC13A2	2.45	X	0.1
ATP6V0A4	1.61	X	0
AVP	1.50	X	0
CLCA4	1.64	X	0
CREG2	1.53	X	0
DEFB121	1.75	X	0
FAM133A	1.59	X	0
LILRA1	1.61	X	0
OR2G3	1.77	X	0
OR51M1	1.72	X	0
OR5AP2	1.77	X	0
WBSCR28	1.69	X	0
ACVR1B	1.66		

ADCK2	1.54		
ADRBK1	1.87		
CCDC96	2.08		
CLK3	1.74		
CSF1R	1.93		
FGFR3	1.59		
FUK	2.32		
IRAK3	1.58		
JAK3	2.12		
LAG3	2.05		
MAP3K9	2.40		
MASTL	1.89		
NAPSA	1.60		
NEK8	2.01		
NME3	1.70		
PCSK9	1.56		
PDK1	2.08		
PIP5K1A	1.61		
PLCG2	2.13		
PSMB1	1.94		
PTGER1	2.09		
PTK2B	2.99		
RPL17	1.87		
SRC	1.81		
STK24	1.61		
STK35	1.87		
TEX264	1.50		
TLK2	1.59		
ULK4	1.74		
WDR1	1.73		
XRN2	1.78		
**Controls**			
KRT80	0.5		
CPA1	0.5		

* FANTOM5 data were used given the apparent problem with HPA RNAseq data for the gene in all tissues

Supplementary Table 2. Box plot details from Fig. 9b-d. T: Tumor. NT: Non-tumor

	Fig. 9b			Fig. 9c			Fig. 9d			
	GSE83148			GSE65359			GSE14520		GSE65485	
	Healthy	HBV DNA (-)	HBV DNA (+)	Tolerance	Clearance	Inactive	NT	T	NT	T
N. of values	6	32	90	22	50	11	198	198	5	50
Minimum	13.66	14.97	12.37	6.84	6.21	6.37	16.74	18.45	1.99	0.72
25% Percentile (lower bound)	14.34	25.33	32.53	7.32	7.62	6.89	22.20	32.09	2.06	5.16
Median	19.80	31.12	48.58	7.59	8.12	7.41	24.52	48.65	2.63	7.50
75% Percentile (upper bound)	26.44	45.96	78.11	7.94	8.40	7.68	29.20	75.15	3.39	15.90
Maximum	30.11	100.20	186.10	8.32	9.15	8.22	47.18	213.30	3.49	42.56

Supplementary Table 3. Box plot details from Fig. 9e and Supplementary Fig. 6b. T: Tumor. NT: Non-tumor

	Fig. 9e								Supplementary Fig. 6b			
	Alc.		HBV		HCV		NAFLD		GSE84044			
	T	NT	T	NT	T	NT	T	NT	F0	F1	F2	F3
Number of values	70	8	76	7	34	5	11	2	37	33	34	15
Minimum	0.72	0.20	1.12	0.66	0.72	0.82	0.79	0.51	3.58	3.76	3.56	4.33
25% Percentile (lower bound)	1.77	0.36	2.23	0.76	2.45	0.86	1.03	0.51	4.49	4.42	4.93	5.24
Median	3.28	0.78	4.09	0.87	4.93	0.98	2.30	0.53	4.93	4.85	5.51	5.71
75% Percentile (upper bound)	8.37	0.91	7.89	0.98	9.37	1.12	5.18	0.56	5.44	5.40	5.93	6.22
Maximum	30.67	1.10	24.82	1.08	21.79	1.19	12.23	0.56	5.96	6.04	6.65	6.78
