# Supplementary material for: A genome-wide gain-of-function screen identifies CDKN2C as a HBV host factor
Source: Nat Commun. 2020 Jun 1;11:2707. doi: 10.1038/s41467-020-16517-w (PMC7264273; doi:10.1038/s41467-020-16517-w)
Supplement: Supplementary file 2 — Reporting Summary [file 41467_2020_16517_MOESM2_ESM.pdf]

nature research

Corresponding author(s): Prof. Thomas Baumert and Dr Eloi Verrier

Last updated by author(s): Apr 24, 2020

Reporting Summary

Nature Research wishes to improve the reproducibility of the work that we publish. This form provides structure for consistency and transparency in reporting. For further information on Nature Research policies, see Authors & Referees and the Editorial Policy Checklist .

Statistics

For all statistical analyses, confirm that the following items are present in the figure legend, table legend, main text, or Methods section.

n/a	Confirmed	
☐	☒	The exact sample size (n) for each experimental group/condition, given as a discrete number and unit of measurement
☐	☒	A statement on whether measurements were taken from distinct samples or whether the same sample was measured repeatedly
☐	☒	The statistical test(s) used AND whether they are one- or two-sided<br>*Only common tests should be described solely by name; describe more complex techniques in the Methods section.*
☒	☐	A description of all covariates tested
☒	☐	A description of any assumptions or corrections, such as tests of normality and adjustment for multiple comparisons
☐	☒	A full description of the statistical parameters including central tendency (e.g. means) or other basic estimates (e.g. regression coefficient) AND variation (e.g. standard deviation) or associated estimates of uncertainty (e.g. confidence intervals)
☐	☒	For null hypothesis testing, the test statistic (e.g. F, t, r) with confidence intervals, effect sizes, degrees of freedom and P value noted<br>*Give P values as exact values whenever suitable.*
☒	☐	For Bayesian analysis, information on the choice of priors and Markov chain Monte Carlo settings
☒	☐	For hierarchical and complex designs, identification of the appropriate level for tests and full reporting of outcomes
☒	☐	Estimates of effect sizes (e.g. Cohen's d, Pearson's r), indicating how they were calculated

Our web collection on statistics for biologists contains articles on many of the points above.

Software and code

Policy information about availability of computer code

Data collection	None
Data analysis	GraphPad PRISM 6 for Windows; Microsoft Excel for Microsoft Office 365 ProPlus (version 1911); Affymetrix Expression Console software (version 1.4.1); Image Lab (Bio-Rad, version 5.2.1)

For manuscripts utilizing custom algorithms or software that are central to the research but not yet described in published literature, software must be made available to editors/reviewers. We strongly encourage code deposition in a community repository (e.g. GitHub). See the Nature Research guidelines for submitting code & software for further information.

Data

Policy information about availability of data

All manuscripts must include a data availability statement. This statement should provide the following information, where applicable:

- Accession codes, unique identifiers, or web links for publicly available datasets
- A list of figures that have associated raw data
- A description of any restrictions on data availability

Data availability. The dataset generated in this study, including the results from the gain-of-function primary screen, are available within Supplementary information. Full immunoblots are provided in Supplementary Fig. 8. The microarray dataset is publicly available in the NCBI Gene Expression Omnibus database (accession number GSE132638 [https://www.ncbi.nlm.nih.gov/geo/query/acc.cgi?acc=GSE132638]). The source data underlying Figs 1, 2, 3, 4, 5, 6, 7, and 9 and Supplementary Figs 1, 4, 5 and 6 are provided as a Source Data file. The details of the box plots presented in Fig.9b-e and Supplementary Fig. 6b are presented in Supplementary Table S2 and Supplementary Table S3. The rest of the data is available through the corresponding authors upon reasonable request. The following public databases were used in the study: GSE83148 [https://www.ncbi.nlm.nih.gov/geo/query/acc.cgi?acc=GSE83148]; GSE65359 [https://www.ncbi.nlm.nih.gov/geo/query/acc.cgi?acc=GSE65359]; GSE84044 [https://www.ncbi.nlm.nih.gov/geo/query/acc.cgi?acc=GSE84044]; GSE65485 [https://www.ncbi.nlm.nih.gov/geo/query/acc.cgi?acc=GSE65485]; GSE14520 [https://www.ncbi.nlm.nih.gov/geo/query/acc.cgi?acc=GSE14520]; TCGA-LIHC [https://www.cancer.gov/about-nci/organization/ccg/research/structural-genomics/tcga]. The following public protocol was used: PoolQ [https://portals.broadinstitute.org/gpp/public/resources/

protocols].

Field-specific reporting

Please select the one below that is the best fit for your research. If you are not sure, read the appropriate sections before making your selection.

[x] Life sciences [ ] Behavioural & social sciences [ ] Ecological, evolutionary & environmental sciences

For a reference copy of the document with all sections, see nature.com/documents/nr-reporting-summary-flat.pdf

Life sciences study design

All studies must disclose on these points even when the disclosure is negative.

Sample size	No sample-size calculation was performed. Minimal sample size was mostly n=6 per group from mostly three independent experiments to allow to derive non-parametric statistical analyses from the data. When n<5 (Fig. 5c notably), no statistical analysis was performed.
Data exclusions	None
Replication	Individual experiments were reproduced three times in an independent manner with similar results except otherwise stated.
Randomization	Not relevant for our study: only one variable is tested in each experiment
Blinding	Not relevant for our study: only one variable is tested in each experiment

Reporting for specific materials, systems and methods

We require information from authors about some types of materials, experimental systems and methods used in many studies. Here, indicate whether each material, system or method listed is relevant to your study. If you are not sure if a list item applies to your research, read the appropriate section before selecting a response.

Materials & experimental systems

n/a	Involved in the study	
[ ]	[x]	Antibodies
[ ]	[x]	Eukaryotic cell lines
[x]	[ ]	Palaeontology
[x]	[ ]	Animals and other organisms
[ ]	[x]	Human research participants
[x]	[ ]	Clinical data

Methods

n/a	Involved in the study	
[x]	[ ]	ChIP-seq
[ ]	[x]	Flow cytometry
[x]	[ ]	MRI-based neuroimaging

Antibodies

Antibodies used	Mouse monoclonal antibody targeting HBsAg (Bio-Techne, clone 1044/329); In-house anti-hepatitis delta antigen (HDAg) antibody purified from serum of an HBV/HDV co-infected patient as described (Verrier et al., Hepatology 2016;63(1):35-48); Rabbit monoclonal anti-CDKN2C antibody (anti-p18 INK4c, ab192239, Abcam); Rabbit polyclonal anti-β-tubulin antibody (GTX101279, Gentex); Rabbit polyclonal anti-GAPDH (ab9485, Abcam); Alexa Fluor® 647 AffiniPure Goat Anti-Mouse IgG (H+L) (Jackson Research 115-605-003); Alexa Fluor® 647-AffiniPure Goat Anti-Human IgG (H+L) (Jackson Research 109-605-003); Anti mouse IgG, Horseradish Peroxidase linked whole antibody (from sheep) (GE HEALTHCARE NA931); Peroxidase-AffiniPure Goat Anti-Rabbit IgG (H+L) (Jackson Research 111-035-144). Antibody dilutions are indicated in the Methods section of the main manuscript
Validation	HBsAg recognizes the Hepatitis B surface antigen. According to the manufacturer's information, the antibody was validated in a ELISA assay using Biogenesis Test Groups (BTGs) showing reactivity with Hepatitis B surface antigen only. The described application is ELISA and immunoflorescence. Moreover, the antibody specificity was validated by IF by our group in the following manuscripts, comparing infected and HBV-infected samples: Verrier et al., Hepatology 2018;68(5):1695-1709<br>HDAg is purified from serum of an HBV/HDV co-infected patient and validated by IF by our group in the following manuscripts comparing HDV-infected and and non-infected samples: Verrier et al., Cell Rep 2016;17(5):1357-1368, Verrier et al., Hepatology 2016;63(1):35-48 and Verrier et al., Gut 2020;69(1):158-167<br>According to the manufacturer's information, the rabbit monoclonal anti-CDKN2C antibody (anti-p18 INK4c, ab192239, Abcam) is suitable for WB, IP, ICC and IHC and reacts with mouse, human and rat CDKN2C. The specificity of the antibody was tested by Western Blot using a KO-CDKN2C control sample. For more information about the antibody: https://www.abcam.com/p18-ink4ccdkn2c-antibody-epr15891-ab192239.html#description_images_3.<br>According to the manufacturer's information, the rabbit polyclonal anti-β-tubulin antibody (GTX101279, Gentex) is suitable for WB, ICC/IF, IHC and reacts with mouse, human, rat and hamster β-tubulin. For more information about this antibody: https://www.genetex.com/Product/Detail/beta-Tubulin-antibody/GTX101279

According to the manufacturer's information, the rabbit polyclonal anti-GAPDH antibody is suitable for IP, ELISA, WB, IHC-Fr, ICC/IF, Flow Cyt and reacts with Mouse, Rat, Chicken, Dog, Human, Saccharomyces cerevisiae, Xenopus laevis, Schizosaccharomyces pombe, African green monkey GAPDH. for more information : https://www.abcam.com/gapdh-antibody-loading-control-ab9485.html

Eukaryotic cell lines

Policy information about cell lines

Cell line source(s)	HepG2 cells were provided by ATCC (HB-8065); The NTCP-overexpressing HepG2 cell line was produced by U1110 laboratory as described (Verrier et al., Hepatology 2016;63(1):35-48); The Huh7 cell line was provided by J. Taylor (Fox Chase Cancer Center, Philadelphia, USA) and available through JCRB (JCRB0403); The NTCP-overexpressing clonal Huh106 cell line was produced from Huh7 cells by C. Sureau (INTS, Paris, FRA) as described (Verrier et al., Hepatology 2016;63(1):35-48). HEK293T cells were provided by B. Haller from the cell culture service of IGBMC (Illkirch, FRA) and available through ATCC (ATCC® CRL-3216™). HepAD38 cells were provided by E. Hildt (Paul-Ehrlich-Institut, Langen, GER).
Authentication	None of the cell lines were authenticated
Mycoplasma contamination	All the cell lines were mycoplasma negative (mycoplasma contamination is routinely controlled every 50 days using PlasmoTest™ - Mycoplasma Detection Kit, InvivoGen rep-pt1)
Commonly misidentified lines (See ICLAC register)	None

Human research participants

Policy information about studies involving human research participants

Population characteristics	Patients with chronic HBV infection. HBV viral load is the only parameter that was used in the study.
Recruitment	Human serum from patients with chronic HBV/HDV infection followed at the Strasbourg University Hospitals, Strasbourg, France was obtained with informed consent. PHH were obtained from liver tissue from patients undergoing liver resection for liver metastasis at the Strasbourg University Hospitals with informed consent. Human samples from HBV infected patients followed at the Chang Gung Memorial Hospital (Taipei, Taiwan) were obtained with informed consent. No self-selction bias or other bias.
Ethics oversight	Protocols were approved by the local Ethics Committee of the Strasbourg University Hospitals (CPP), the Ministry of Higher Education and Research of France (DC-2016-2616), and the local Ethics Committee from the Chang Gung Memorial Hospital (Taipei, Taiwan) (Institutional Review Board 102-3825C).

Note that full information on the approval of the study protocol must also be provided in the manuscript.

Flow Cytometry

Plots

Confirm that:

- [x] The axis labels state the marker and fluorochrome used (e.g. CD4-FITC).
- [x] The axis scales are clearly visible. Include numbers along axes only for bottom left plot of group (a 'group' is an analysis of identical markers).
- [x] All plots are contour plots with outliers or pseudocolor plots.
- [x] A numerical value for number of cells or percentage (with statistics) is provided.

Methodology

Sample preparation	Cells were fixed in 100% methanol for at least 20 minutes at -20 ºC. Cells were then blocked and permeabilized using PBS 1% FBS, 0.05% saponin for 30 min at RT. HBsAg was stained using a mouse monoclonal anti-HBsAg Ab (Bio-Techne, clone 1044/329) for 30 minutes at 4 ºC and then with an AF647-labelled secondary antibody targeting mouse IgGs (Jackson Research) for 30 minutes at 4 ºC. For flow cytometry analysis of DNA content, cells were fixed in ice-cold 75% ethanol in water for 30 minutes at 4ºC. Cells were washed and resuspend and incubated in PBS 50 µg/mL propidium iodide (Invitrogen) and 50 µg/mL Ribonuclease A (Sigma) for 30 min at RT. Cells were subsequently washed and resuspended in PBS 5 µM EDTA
Instrument	The screen were performed on a FACSARIA III (BD). The other flow cytometry analysis were performed using a CYTOFLEX (Beckman Coulter).
Software	The sorting were analyse with FACSDIVA from BD and the flow cytometry analysis were analysed using the software CytExpert from Beckman Coulter
Cell population abundance	20 millions of cells were sorted. A post-sort control were performed by an analysis of the post-sort fraction.

Gating strategy	The gating was first performed using a FCS/SSC dot plot. The gain used was fixed when all populations was observed on the plot. The main population was gated to perform a singlets plot using FSC-H and FSC-A parameters. The histogram plot and the dot plots were created from the singlets gate using Count/APC-A or SSC-A/APC parameters. The gating strategy is presented in Supplementary Figure 7.

☒ Tick this box to confirm that a figure exemplifying the gating strategy is provided in the Supplementary Information.
